# Heritability and Genome-Wide Association Study of Plasma Cholesterol in Chinese Adult Twins

**DOI:** 10.3389/fendo.2018.00677

**Published:** 2018-11-15

**Authors:** Hui Liu, Weijing Wang, Caixia Zhang, Chunsheng Xu, Haiping Duan, Xiaocao Tian, Dongfeng Zhang

**Affiliations:** ^1^Department of Epidemiology and Health Statistics, Public Health College, Qingdao University, Qingdao, China; ^2^Qingdao Municipal Centre for Disease Control and Prevention, Qingdao, China

**Keywords:** cholesterol, dyslipidemias, genetics, GWAS, heritability, HDL, LDL, twins

## Abstract

Dyslipidemia represents a strong and independent risk factor for cardiovascular disease. Plasma cholesterol, such as total cholesterol (TC), low density lipoprotein cholesterol (LDL-C), and high density lipoprotein cholesterol (HDL-C), is the common indicator of diagnosing dyslipidemia. Here based on 382 Chinese twin pairs, we explored the magnitude of genetic impact on TC, HDL-C, LDL-C variation and further searched for genetic susceptibility loci for them using genome-wide association study (GWAS). The ACE model was the best fit model with additive genetic parameter (A) accounting for 26.6%, common or shared environmental parameter (C) accounting for 47.8%, unique/non-shared environmental parameter (E) accounting for 25.6% for the variance in HDL-C. The AE model was the best fit model for TC (A: 61.4%; E: 38.6%) and LDL-C (A: 65.5%; E: 34.5%). While no SNPs reached the genome-wide significance level (*P* < 5 × 10^−8^), 8, 14, 9 SNPs exceeded the suggestive significance level (*P* < 1 × 10^−5^) for TC, HDL-C, LDL-C, respectively. The promising genetic regions for TC, HDL-C, LDL-C were on chromosome 11 around rs7107698, chromosome 5 around rs12518218, chromosome 2 around rs10490120, respectively. Gene-based analysis found 1038, 1033 and 1090 genes nominally associated with TC, HDL-C, LDL-C (*P* < 0.05), especially *FAF1, KLKB1* for TC, *KLKB1* for HDL-C, and *NTRK1, FAF1, SNTB2* for LDL-C, respectively. The number of common related genes among TC, HDL-C and LDL-C was 71, including *FAF1, KLKB1*, etc. Pathway enrichment analysis discovered known related pathways-zinc transporters, metal ion SLC transporters for TC, cell adhesion molecules CAMs, IL-6 signaling for HDL, FC epsilon RI signaling pathway, NFAT pathway for LDL, respectively. In conclusion, the TC and LDL-C level is moderately heritable and the HDL-C level is lowly heritable in Chinese population. The genomic loci, functional genes and pathways are identified to account for the heritability of plasma cholesterol level. Our findings provide important insights into plasma cholesterol molecular physiology and expect future research to replicate and validate our results.

## Introduction

Dyslipidemia represents a strong and independent risk factor for cardiovascular disease (1), a leading cause of death worldwide (2, 3). Besides, many studies indicate that dyslipidemia can increase the risk of preeclampsia, colorectal cancer, diabetic macular edema and polycystic ovary syndrome (4–7). The prevalence of dyslipidemia is high and is increasingly prevalent in China (8, 9). Plasma cholesterol, such as total cholesterol (TC), low density lipoprotein cholesterol (LDL-C), high density lipoprotein cholesterol (HDL-C), is a common indicator of diagnosing dyslipidemia. Therefore, exploring factors affecting plasma cholesterol homeostasis is a crucial step toward providing early prevention and therapeutic targets for dyslipidemia.

The plasma cholesterol level is mediated by a combination of genetic and environmental factors. At present, the heritability for plasma cholesterol level has been estimated in several studies, with the heritability of TC, HDL-C, LDL-C level ranging from 0 to 89% (10–13), 22 to 93% (13–16), and 22 to 91% (10, 13, 16, 17), respectively. Additionally, several genome-wide association studies (GWAS) have attempted to find susceptible genetic loci located in the corresponding gene affecting plasma cholesterol level. A GWAS conducted by Middelberg RP et al. (18) for plasma cholesterol found genes *LPL, LIPC, CETP* associated with HDL-C level and *CELSR2, APOB, TOMM40* with LDL-C level. Another two GWAS studies identified *SRGAP2, HOXC13* and *CD47, DUSP4* that were associated with HDL-C and TC level, respectively (19, 20).

However, the known genetic variations only explain a small proportion of the genetic contribution and many potential genetic genes and loci remain to be discovered. Besides, allele frequencies, life style and environmental contributions differ between Chinese and other ethnic populations. Twin samples will have a higher power in genetic study, especially in human complex diseases (21). Here based on 382 Chinese twin pairs, we explored the genetic effect on TC, HDL-C, LDL-C variation and further searched for genetic susceptibility loci for these traits using GWAS (22).

## Materials and methods

### Twin samples collection and phenotypic measurement

The twin samples were collected from Qingdao Twin Registry and the details can be found in the literatures (23, 24). Twins who were pregnancy and lactation, as well as incomplete co-twin pairs were excluded. Twin pairs that taking cholesterol-lowering drugs also were excluded. The study included 382 twin pairs for heritability analysis and 139 dizygotic (DZ) twin pairs for GWAS with a mean age of 51.6 ± 7.7. All twin samples undertook the blood sampling and a physical examination after a 10–12 h overnight fast and completed a questionnaire. The zygosity of same sex and blood type was identified by using 16 multiple short tandem sequence repeat DNA markers (25, 26). According to the standard procedure using an automatic biochemical analyzer (Hitachi 7600; Hitachi, Tokyo, Japan), we tested participants' fasting blood cholesterol sample in the Qingdao Diabetes Hospital. Friedewald equation was used to calculate the LDL-C level: LDL-C (mmol/L) = TC–HDL-C–(TG/2.2) (mmol/L) (27).

The Regional Ethics Committee of the Qingdao CDC Institutional Review Boards approved this study and the ethical principles followed the Helsinki Declaration. All subjects signed the written informed consent.

### Genotyping, imputation, and quality control

Genome-wide SNPs were genotyped using the Illumina's InfiniumOmni2.5Exome-8v1.2 Bead-Chip platform. We performed stringent genotype quality control procedures: locus missing (< 0.05), minor allele frequency (MAF > 0.05), call rate (>0.98), and Hardy-Weinberg Equilibrium (HWE > 1 × 10^−4^). The final number of SNPs included in the subsequent GWAS analysis was 1,365,181.

We used the IMPUTE2 (28) software to impute un-typed SNPs using the LD information from 1,000 Genomes Project Phase 3 reference panel (29) (CHS *N* = 171 and CHB *N* = 142). We used *R*^2^ > 0.6, MAF > 0.05 and HWE > 1 × 10^−4^ to filter the imputed data, and the 7,405,822 SNPs were used to explore the association with plasma cholesterol level.

### Statistical analysis

#### Heritability

Data preparation and statistical description were performed with SPSS version 22.0. We used the structural equation models (SEM) to evaluate the genetic variance components with Mx software^1^. The Blom's formula was used to guarantee the normal distribution or approximate normal distribution because of the deviation of the distribution of all indicators. Pearson's product-moment correlation coefficient was used to calculate intra-class phenotypic correlations. The correlation in MZ twins was significantly higher than DZ twins, reflecting the importance of genetic effects in plasma cholesterol levels.

The total phenotypic variance could be decomposed to additive genetic variance (A), common or shared environmental variance (C), and unique/non-shared environmental variance (E). The full ACE model was firstly fitted, the likelihood-ratio χ^2^-test was applied to test whether the contributions of A or C to the model had statistical significance by comparing the full model (ACE) and their nested models (CE and AE). The Akaike information criterion (AIC), which is equal to the goodness-of-fit χ^2^-value minus twice the degrees of freedom, was used to indicate the parsimony of each model and a lower AIC indicated a better fit. Heritability (h^2^) was calculated in the best-fitting model based on the ratio of additive genetic variation to total phenotypic variation, with adjusting for the effect of age, sex and education level. We calculated the power of twin pairs for additive genetic influences by Mx software, and the calculation results showed that the power of our heritability analysis was above 90%.

#### GWAS

##### SNPs-based analysis

We used the genome-wide efficient mixed-model association (GEMMA) to test the association between plasma cholesterol level and SNP genotypes after adjusting for the following covariates: sex, age, and education level (30). We used Quantile-quantile (Q-Q) and Manhattan plots to illustrate overall significance level (*P* < 5 × 10^−8^) and suggestive level (*P* < 1 × 10^−5^) autosomal and chromosomal X SNPs (31). In addition, we analyzed the enrichment of cell-type enhancers for the typed GWAS results of the regulatory domains outside the coding regions by using online HaploReg v4.1 software^2^ (32, 33). SNPs with *P* < 1 × 10^−5^ were selected as query SNPs, and an uncorrected *P* < 0.05 for enrichments of cell-type enhancers were reported.

##### Gene-based analysis

The gene-based analysis integrated all SNPs within a gene to increase the signal or strength of association, which correcting for linkage disequilibrium (LD) and gene size. The gene-based test was implemented in Versatile Gene-based Association Study-2 (VEGAS2)^3^ which uses 1000 Genomes data to simulate the correlations of SNPs across the autosomes and chromosome X. (34, 35). We used SNPs from “1000G East ASIAN Population”. The genome-wide significant gene for the association was defined as *P* < 2.63 × 10^−6^ (0.05/19,001) as 19,001 genes being evaluated.

##### pathway enrichment analysis

We used PASCAL to compute pathway-scored (36–38). In this approach, genetic markers SNPs were first mapped to genes, and the association scores of all genes in the pathway were computed. We then combined the genes scores of the same pathways to calculate the pathway scores. We used chi-squared or empirical score to evaluate pathway enrichment of high-scoring (possibly fused) genes, avoiding any standard binary enrichment tests with inherent *P*-value thresholds. Pathways and their corresponding gene annotation were obtained from KEGG, Reactome, and Biocarta (as defined in MSigDB)^4^.

## Results

### Heritability

The final sample consisted of 382 twin pairs for heritability analysis and 139 DZ twin pairs for GWAS analysis with a mean age of 51.6 ± 7.7. The mean value ± SD of TC, HDL-C, LDL-C level for all subjects was 4.9 ± 1.2 umol/L, 1.5 ± 0.5 umol/L, 2.8 ± 0.9 umol/L, respectively (Supplemental Table 1). MZ twin correlations for TC (rMZ = 0.61, 95%CI: 0.52–0.68), HDL-C (rMZ = 0.74, 95%CI: 0.68–0.79), LDL-C (rMZ = 0.65, 95%CI: 0.57–0.72) level were all larger than DZ twin correlations (rDZ = 0.35, 95%CI: 0.21–0.47; rDZ = 0.61, 95%CI: 0.50–0.70; rDZ = 0.35, 95%CI: 0.21–0.46), respectively, indicating the existence of genetic effects (Supplemental Table 2). The full ACE model was first determined and then the likelihood ratio test and AIC was applied to choose the nested models. Finally, the ACE model was the best fit model with A accounting for 26.6% (95% CI: 7.9–48.9), C accounting for 47.8% (95% CI: 26.3–64.6), E accounting for 25.6% (95% CI: 20.8–31.6) for the variance in HDL-C level. The best fit model for TC level was AE model with A accounting for 61.4% (95% CI: 53.2–68.3), E accounting for 38.6% (95% CI: 31.7–46.8) and for LDL-C level was also AE model with A accounting for 65.5% (95% CI: 57.9–71.8), E accounting for 34.5% (95% CI: 28.2–42.2) (Table 1).

**Table 1:** Model fit and proportion of variance for TC, HDL-C, LDL-C level accounted by genetic and environmental parameters.

**Variable**	**Model**	**A (95%CI)**		**C (95%CI)**		**E (95%CI)**		**−2LL**	**df**	**AIC**	**χ^2^**	***P***
TC	ACE	51.8	(23.6–67.9)	9.0	(0–33.7)	39.2	(32.0–47.9)	1996.9	757	482.9	
	CE	–	–	49.5	(41.5–56.8)	50.5	(43.2–58.5)	2010.3	758	494.3	13.4	2.49E−04
	AE^*^	**61.4**	**(53.2–68.3)**	**–**	**–**	**38.6**	**(31.7–46.8)**	**1997.4**	**758**	**481.4**	**0.4**	**5.13E**−**01**
HDL-C	ACE^*^	**26.6**	**(7.9–48.9)**	**47.8**	**(26.3–64.6)**	**25.6**	**(20.8–31.6)**	**1763.6**	**757**	**249.6**	
	CE	–	–	69.4	(63.7–74.4)	30.6	(25.6–36.3)	1771.7	758	255.7	8.1	4.48E−03
	AE	74.9	(69.5–79.4)	–	–	25.1	(20.6–30.5)	1778.5	758	262.5	14.9	1.15E−04
LDL-C	ACE	61.5	(34.9–71.7)	3.8	(0–27.4)	34.7	(28.3–42.7)	2028.1	760	508.1	
	CE	–	–	50.9	(43.0–58.0)	49.1	(42.0–57.0)	2049.4	761	527.4	21.2	4.06E−06
	AE^*^	**65.5**	**(57.9–71.8)**	**–**	**–**	**34.5**	**(28.2–42.2)**	**2028.2**	**761**	**506.2**	**0.09**	**7.71E**−**01**

* *, The best fitted model; −2LL, −2 Log Likelihood; df, degree of freedom; χ^2^, difference of χ^2^-value; P, χ^2^ test in model fitting; A, additive genetic influence; AIC, Akaike's information criterion; C, common or shared environmental influence; E, unique or non-shared environmental influence; HDL-C, high density lipoprotein cholesterol; LDL-C, low density lipoprotein cholesterol; TC, total cholesterol. The content discussed in detail were in bold*.

### GWAS

#### SNPs-based analysis

##### TC level

A sample of 139 DZ twin pairs including 1,365,181 qualified SNPs was included for the present GWAS. The Q-Q plot about TC level illustrated the relationship between the observed and expected GWAS *P*-values (Supplemental Figure 1). The genomic inflation factor (λ-statistic = 1) revealed no evidence of inflation of the test statistics due to population stratification. And weak association was shown due to the slight deviation in the upper right tail from the null distribution. No SNPs reached the genome-wide significance level (*P* < 5 × 10^−8^) as illustrated in Manhattan plot (Supplemental Figure 2). However, there were 8 SNPs exceeding the threshold for suggestive significance level (*P* < 1 × 10^−5^) (Supplemental Table 3). The strongest association SNP was rs7107698 (*P* = 2.29 × 10^−6^). As the locus zoom plots illustrated, one chromosomal loci 11p15.4 showed nominal association with TC level (Supplemental Figure 3). Four SNPs (*P* = 2.29 × 10^−6^ to 2.45 × 10^−6^) were located at *AMPD3* gene on chromosome 11p15.4. By HaploReg v4.1, three cell-type specific enhancers (uncorrected *P* < 0.05) of primary neutrophils from peripheral blood (*P* = 0.04), ovary (*P* = 0.04) and NHDF-Ad adult dermal fibroblast primary cells (*P* = 0.01) were identified for TC level (Supplemental Table 4).

##### HDL-C level

**Figure 1:**
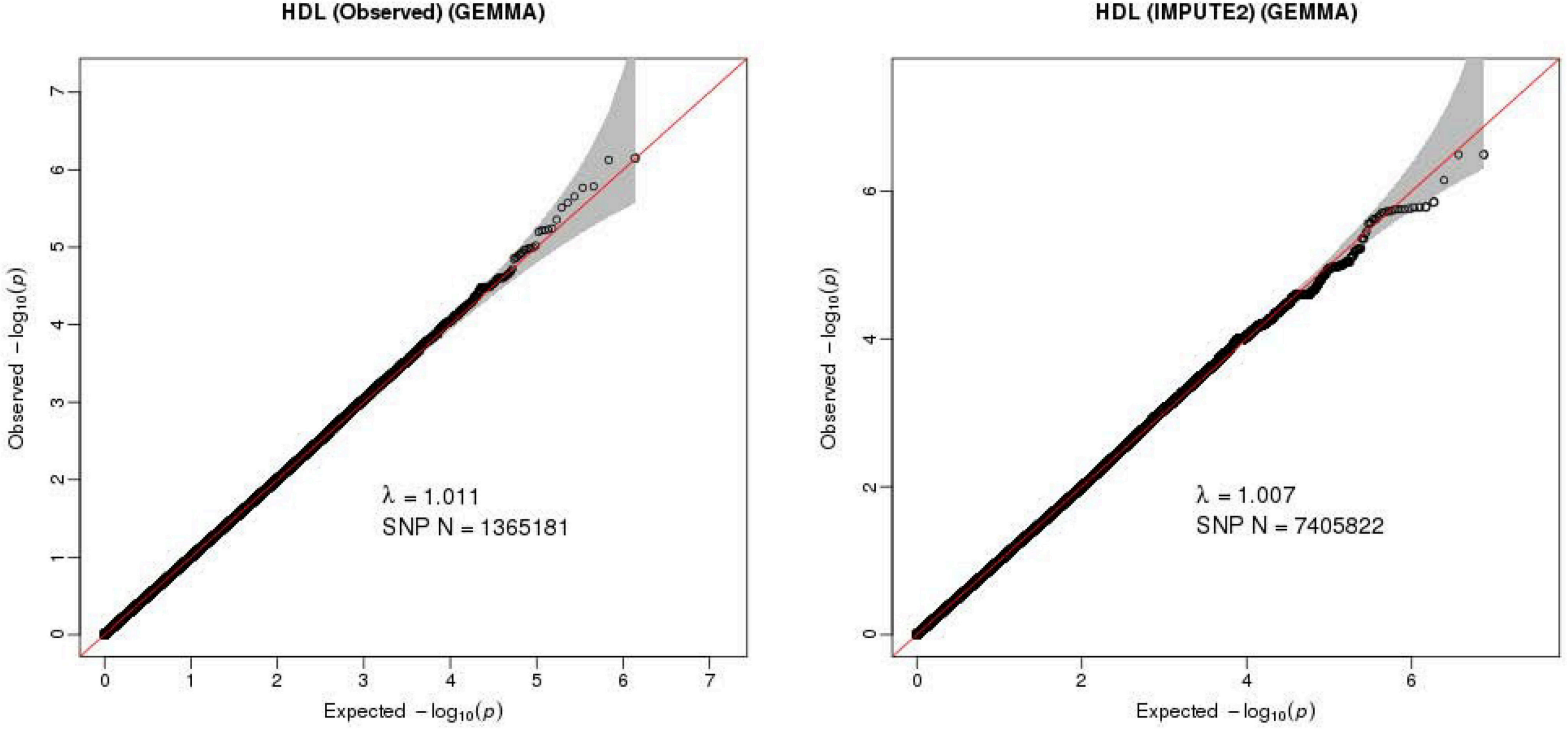
Quantile-quantile plot for quality control check and visualizing crude association for genome-wide association study of HDL-C level. The x-axis shows the –log10 of expected *P*-values of association from chi-square distribution and the y-axis shows the –log10 of *P*-values from the observed chi-square distribution. The black dots represent the observed data, and the red line is the expectation under the null hypothesis of no association.

**Figure 2:**
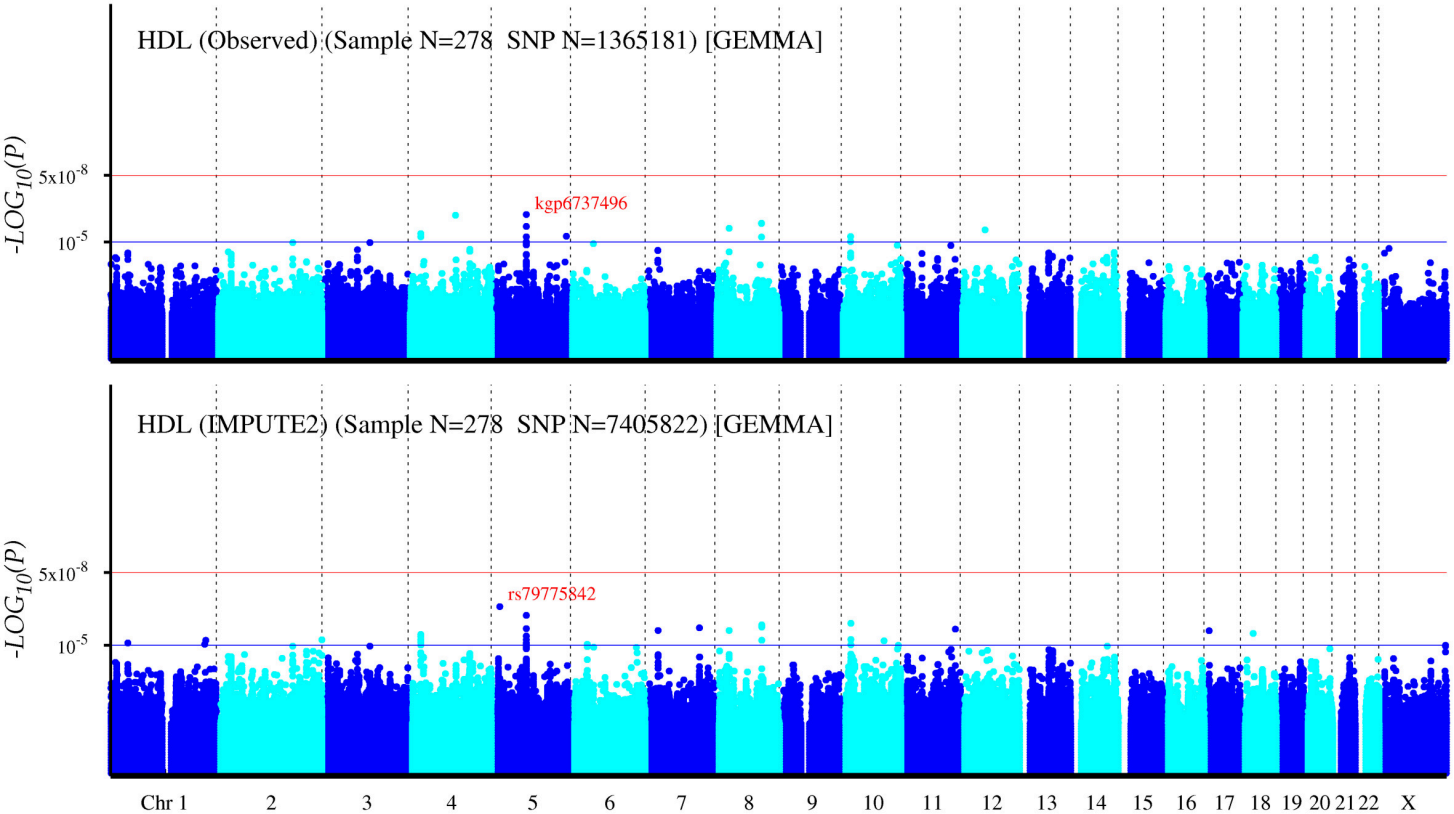
Manhattan plot for genome-wide association study of HDL-C level. The x-axis shows the numbers of autosomes and the X chromosome, and the y-axis shows the –log10 of *P*-values for statistical significance. The dots represent the SNPs. None of the SNPs reached the genome-wide significance level (*P* < 5 × 10^−8^).

**Figure 3:**
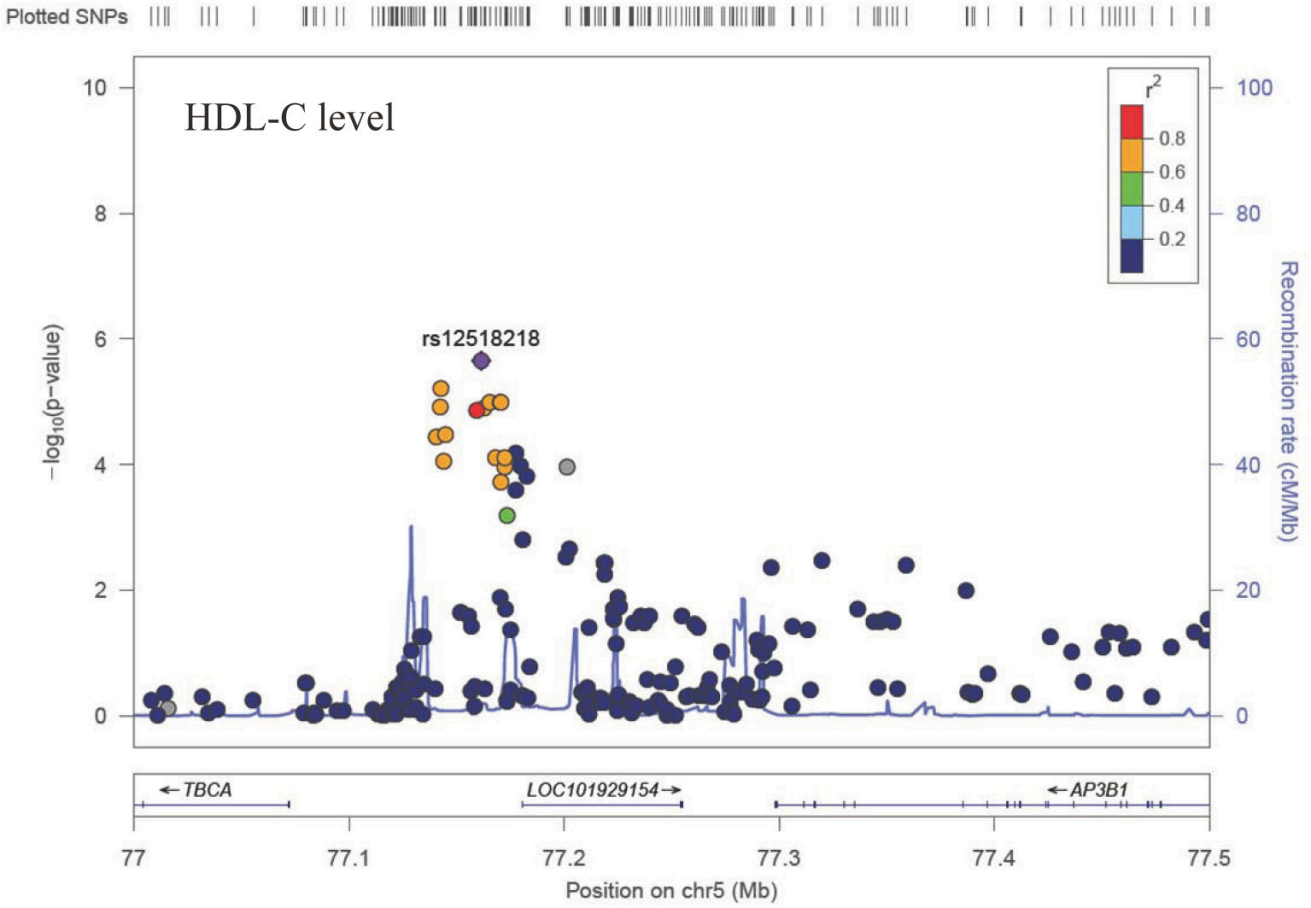
Regional association plot showing signal around chromosomal loci of 5q14.1 for genome-wide association study of HDL-C level.

The Q-Q plot about HDL-C level was shown in Figure 1. None of the SNPs reached the genome-wide significance level (*P* < 5 × 10^−8^) as illustrated in Manhattan plot (Figure 2). However, there are 14 SNPs exceeding the threshold for suggestive significance level (*P* < 1 × 10^−5^) (Table 2). The strongest association SNP was kgp6737496 (rs199929635) (*P* = 7.10 × 10^−7^). Chromosomal loci 5q14.1 showed suggestive association with HDL-C level, which including kgp6737496 (rs199929635), rs12518218, rs7729225 near *LOC101929154* genes (Figure 3).

**Table 2:** The summary of SNPs with *P* < 1 × 10^−5^ for association with HDL-C in typed GWAS data.

**SNP**	**Chr band**	**CHR**	**BP**	***P*-value**	**Closest genes or genes**	**Official full name**
**kgp6737496** (rs199929635)	5q14.1	5	77,142,788	7.10E-07	*LOC101929154*	Uncharacterized LOC101929154
rs28402213	4q24	4	106,517,449	7.51E-07	*ARHGEF38*	Rho guanine nucleotide exchange factor 38
rs6468909	8q22.3	8	105,227,985	1.65E-06	*RIMS2*	Regulating synaptic membrane exocytosis 2
rs72685070	8q22.3	8	105,250,237	1.71E-06	*RIMS2*	Regulating synaptic membrane exocytosis 2
**rs12518218**	5q14.1	5	77,161,489	2.22E-06	*LOC101929154*	Uncharacterized LOC101929154
rs241178	8p21.1	8	28,626,418	2.67E-06	*INTS9*	Integrator complex subunit 9
rs56207115	12q13.13	12	52,994,896	3.08E-06	*KRT72*	Keratin 72
rs10939012	4p15.2	4	24,897,032	4.43E-06	*CCDC149*	Coiled-coil domain containing 149
rs10053012	5q35.1	5	171,522,275	5.71E-06	*STK10*	Serine/threonine kinase 10
**rs12414709**	10p13	10	17,041,083	5.90E-06	*CUBN*	Cubilin
rs12511068	4p15.2	4	24,896,658	6.04E-06	*CCDC149*	Coiled-coil domain containing 149
**rs7729225**	5q14.1	5	77,142,829	6.05E-06	*LOC101929154*	Uncharacterized LOC101929154
kgp10999245 (rs201647698)	8q22.3	8	105,243,123	6.36E-06	*RIMS2*	Regulating synaptic membrane exocytosis 2
**rs17345993**	10p13	10	17,032,885	9.56E-06	*CUBN*	Cubilin

*kgp, 1,000 Genomes Project; CHR, chromosome. The content discussed in detail were in bold*.

##### LDL-C level

The Q-Q plot about LDL-C level was shown in Supplemental Figure 4. None of the SNPs reached the genome-wide significance level (*P* < 5 × 10^−8^) as illustrated in Manhattan plot (Supplemental Figure 5). However, there are 9 SNPs exceeding the threshold for suggestive significance level (*P* < 1 × 10^−5^) (Supplemental Table 5). The strongest association SNP was rs10490120 (*P* = 1.11 × 10^−6^). At chromosomal loci 2p16.3, four SNPs rs10490120, rs4953640, rs74263479, rs17037869 were positioned closest to *FSHR* gene that was involved in follicle stimulating hormone receptor (Supplemental Figure 6). HUES6 cells (*P* = 0.01), HUES64 cells (*P* = 0.01) and iPS-18 cells (*P* = 0.01) were confirm as cell-type specific enhancers for LDL-C level (Supplemental Table 6).

##### Imputation

To maximize the identification of new risk variants, we imputed typed SNPs using 1,000 Genomes Project Phase 3 as the reference panel. Manhattan plots for all post-imputation variants showed none evidence of genome-wide significance level (*P* < 5 × 10^−8^) (Figure 2; Supplementary Figures 2, 5). However, there were 39, 55, and 59 SNPs exceeding the threshold for suggestive significance level (*P* < 1 × 10^−5^) for TC, HDL-C, and LDL-C, respectively. The strongest associations were rs77348447, rs79775842, rs56047090 for TC, HDL-C, LDL-C, respectively (Supplemental Tables 7–9).

We also compared our post-imputation results with 34,421 East Asians lipids GWAS meta-analysis results^5^ And 32 SNPs located in genes *RGS5, OSBPL10, ADGRB3, PSMB7, SRSF8* for TC level, 50 SNPs located in genes *KAZN, LOC105378657, LOC105373529, LOC105373941, BIN3, LINC02153, LDHAL6CP* for HDL-C level, 39 SNPs located in genes *VPS13D, IGF2BP2, PSMB7, CA10, NECTIN2, APOE, APOC1, TOMM40* for LDL-C level could be replicated (Supplemental Table 10).

#### Gene-based analysis

In the gene-based analysis, none genes were found to achieve genome-wide significance level and the number of genes that were nominally associated with TC, HDL-C, LDL-C level was 1,038, 1,033, and 1,090, respectively (*P* < 0.05). The top 20 genes were ranked by their *P* values (TC: Supplemental Table 11, HDL-C: Table 3, LDL-C: Supplemental Table 12). *LOC101929154, RIMS2, INTS9, KRT72, CCDC149, CUBN* genes for HDL-C level *and AMPD3, ABR* genes for TC level had already been shown in the suggestive level SNPs-based analysis, whereas the other genes were novel.

**Table 3:** The top 20 genes from VEGAS2 gene-based analysis showing the strongest association with HDL-C level (*P* < 0.05) in typed GWAS data.

**Chr**	**Gene**	**Numbers of SNPs**	**Start position**	**Stop position**	**Gene-based test statistic**	**Gene** ***P-*value**	**Top-SNP**	**Top-SNP** ***P*-value**
11	*RRP8*	9	6,621,143	6,624,880	57.80	4.00E-06	rs17834692	1.58E-04
1	*RLF*	23	40,627,040	40,706,593	164.27	1.20E-05	rs16827079	2.88E-05
4	*MGARP*	7	140,187,316	140,201,492	54.60	2.30E-05	rs3208941	2.04E-05
12	*SLC26A10*	5	58,013,692	58,019,934	45.55	2.90E-05	rs923828	1.79E-04
5	*CTXN3*	15	126,984,712	126,994,322	99.49	3.80E-05	rs248709	4.16E-04
3	*UROC1*	39	126,200,007	126,236,616	243.37	4.10E-05	rs1091553	1.99E-04
4	***KLKB1***	19	187,148,671	187,179,625	113.75	7.10E-05	rs1912826	1.31E-04
17	*CBX4*	6	77,806,954	77,813,213	54.62	8.60E-05	rs73422123	7.25E-05
19	*ACPT*	7	51,293,671	51,298,481	58.32	1.00E-04	rs55735528	5.48E-04
20	*CSTL1*	10	23,420,321	23,425,567	52.62	1.30E-04	rs3746737	3.40E-04
1	*ISG15*	5	948,846	949,919	36.63	1.80E-04	rs116002608	8.77E-05
19	*C19orf48*	13	51,300,949	51,308,110	112.72	1.90E-04	rs4801853	2.61E-04
1	*TMCO2*	2	40,713,572	40,717,365	17.20	2.10E-04	rs61200654	3.36E-05
4	*NDUFC1*	4	140,211,070	140,223,705	38.88	2.20E-04	rs12642647	2.49E-05
2	*SOWAHC*	5	110,371,910	110,376,564	43.04	2.40E-04	rs6726252	2.79E-04
19	*ZNF776*	7	58,258,163	58,269,527	45.19	3.10E-04	rs35919456	2.11E-04
1	*CD101*	24	117,544,371	117,579,173	106.37	3.30E-04	rs1555793	1.20E-04
10	*IFIT1B*	3	91,137,812	91,144,962	18.81	4.00E-04	rs10887951	7.36E-04
1	*HECTD3*	6	45,468,219	45,477,027	42.86	4.20E-04	rs7541207	2.66E-04
12	*FBXO21*	10	117,581,584	117,628,300	90.53	4.40E-04	rs2279766	5.40E-04

*Chr, chromosome. The content discussed in detail were in bold*.

The number of common nominally associated genes (*P* < 0.05) between TC VS HDL-C level, TC VS LDL-C level, HDL-C VS LDL-C level was 195, 504, 91, respectively. And 71 common genes were found among the three traits (Supplemental Table 13).

#### Pathway enrichment analysis

PASCAL program was used in the pathway enrichment analysis. The number of pathways that were nominally associated with TC, HDL-C, and LDL-C level was 816, 529, and 813, respectively (*P* < 0.05). The top 20 pathways were ranked by their emp-*P*-values (TC: Supplemental Table 14; HDL-C: Table 4; LDL-C: Supplemental Table 15). The Q-Q plots were shown in Supplemental Figures 7–9. Pathway analysis revealed several biological processes significantly associated with TC level: zinc transporters, metal ion SLC transporters, ERKS ARE inactivated, MAPK targets nuclear events mediated by map kinases, ERK MAPK targets, transport of glucose and other sugars bile salts and organic acids metal ions and amine compounds, amino acid transport across the plasma membrane, etc.

**Table 4:** The top 20 pathway results-KEGG, Reactome, and Biocarta (emp-*P* < 0.05) using PASCAL program for HDL-C level in typed GWAS data.

**Pathway**	**chisq-*P***	**emp-*P***	**-log(chisq*P*)**	**–log(emp*P*)**
REACTOME_CELL_SURFACE_INTERACTIONS_AT_THE_VASCULAR_WALL	7.11E-04	2.94E-04	3.14818	3.53165
BIOCARTA_HER2_PATHWAY	4.39E-04	4.35E-04	3.35736	3.36151
KEGG_**CELL_ADHESION_MOLECULES_CAMS**	6.25E-04	4.44E-04	3.20421	3.35262
REACTOME_**IL_6_SIGNALING**	6.27E-04	4.66E-04	3.20281	3.33161
KEGG_**TIGHT_JUNCTION**	2.91E-03	6.20E-04	2.53649	3.20761
BIOCARTA_AHSP_PATHWAY	8.78E-04	8.80E-04	3.05652	3.05552
REACTOME_**BILE_SALT_AND_ORGANIC_ANION_SLC_TRANSPORTERS**	9.23E-04	9.30E-04	3.03463	3.03152
REACTOME_**TRANSPORT_OF_GLUCOSE_AND_OTHER_SUGARS_BILE** **_SALTS_AND_ORGANIC_ACIDS_METAL_IONS_AND_AMINE_COMPOUNDS**	9.23E-04	9.50E-04	3.03463	3.02228
REACTOME_**SIGNALING_BY_ILS**	1.35E-03	1.17E-03	2.87021	2.93181
BIOCARTA_CHREBP2_PATHWAY	1.13E-03	1.30E-03	2.94743	2.88606
REACTOME_INTRINSIC_PATHWAY	1.51E-03	1.38E-03	2.82086	2.86012
REACTOME_FORMATION_OF_FIBRIN_CLOT_CLOTTING_CASCADE	1.51E-03	1.48E-03	2.82086	2.82974
REACTOME_NEPHRIN_INTERACTIONS	4.77E-03	1.82E-03	2.32139	2.73993
BIOCARTA_**IL6_PATHWAY**	2.04E-03	1.96E-03	2.69087	2.70774
KEGG_**NATURAL_KILLER_CELL_MEDIATED_CYTOTOXICITY**	7.95E-03	2.15E-03	2.09938	2.66756
REACTOME_**CHONDROITIN_SULFATE_BIOSYNTHESIS**	2.36E-03	2.31E-03	2.62634	2.63639
KEGG_**SULFUR_METABOLISM**	2.36E-03	2.34E-03	2.62634	2.63078
KEGG_**GLYCOSAMINOGLYCAN_BIOSYNTHESIS_CHONDROITIN_SULFATE**	2.36E-03	2.39E-03	2.62634	2.6216
REACTOME_APC_C_CDH1_MEDIATED_DEGRADATION_OF_CDC20_AND_OTHER_ APC_C_CDH1_TARGETED_PROTEINS_IN_LATE_MITOSIS_EARLY_G1	5.66E-03	2.61E-03	2.24755	2.58336
KEGG_**LEUKOCYTE_TRANSENDOTHELIAL_MIGRATION**	2.96E-03	2.62E-03	2.52912	2.5817

chisq-P, Chi-square p-value. Chi-squared method (gene-score p-value were ranked and transformed to a uniform distribution, these values were then transformed by a chi-square quantile function, and summed). The content discussed in detail were in bold.

*emp-P, empirical p-value. Empirical sampling method (gene-scores are transformed with chi-square quantile function and summed, then Monte Carlo estimate of the p-values were obtained by sampling random sets of the same size)*.

Several biological pathways significantly related to HDL-C levels emerged: cell adhesion molecules CAMs, IL-6 signaling, tight junction, bile salt and organic anion SLC transporters, transport of glucose and other sugars bile salts and organic acids metal ions and amine compounds, signaling by ILS, IL6 pathway, natural killer cell mediated cytotoxicity, sulfur metabolism, chondroitin sulfate biosynthesis, glycosaminoglycan biosynthesis chondroitin sulfate, leukocyte transendothelial migration, etc.

The pathways associated with LDL-C involved: FC epsilon RI signaling pathway, NFAT pathway, metabolism of amino acids and derivatives, ERBB signaling pathway, IL1R pathway, HIV infection, etc.

## Discussion

### Heritability

Our investigation was based on 382 twin pairs to explore the heritability of TC, HDL-C, LDL-C level, and confirmed the genetic variants underlying these trait by GWAS. The correlation in MZ twins was significantly higher than DZ twins, which reflected the existence of genetic variance in TC, HDL-C, LDL-C levels (Supplemental Table 2). The ACE model was the best fit model with A accounting for 26.6%, C accounting for 47.8%, E accounting for 25.6% for the variance in HDL-C level. The best fit model for TC level was AE model with A accounting for 61.4%, E accounting for 38.6% and for LDL-C level was also AE model with A accounting for 65.5%, E accounting for 34.5% (Table 1). The magnitude of the additive genetic components (A) for TC level obtained here was in line with several previous twin studies (15, 16, 27, 39–47). HDL-C level is close to some other twin studies (11, 14, 27, 48, 49). LDL-C level is similar to the others (27, 42, 43, 45, 47, 50).

### GWAS

#### SNP-based analysis

##### TC level.

While no genome-wide significant SNPs were found in GWAS for TC level, one SNP rs7107698 on chromosome 11 was found as promising genetic regions. The *AMPD3* gene around the rs7107698 has been linked to the TC level. Adenosine monophosphate deaminase 3 (*AMPD3*) is an enzyme that catalyzes the hydrolytic deamination of adenosine monophosphate to inosine monophosphate and participates in the pathway of adenylate catabolic (51). The activation of AMP deaminase leads to the increase of uric acid and the production of mitochondrial oxidative stress, which further stimulates the formation of de novo lipogenesis and the activation of ATP-citrate lyase and the synthesis of long-chain saturated fatty acids and eventually affects TC level (52, 53). Additionally, three highly correlated SNPs (rs4909928, rs12184411, kgp6520322) were detected within *AMPD3* gene, showing suggestive evidence of association with TC level.

##### HDL-C level.

This cluster of suggestive SNPs (kgp6737496, rs12518218, rs7729225) is linked to *LOC101929154*, a non-coding RNA. Although we have not fully elucidated the biological function of this ncRNA, mutations in base pairs may affect their regulatory domain to regulate HDL-C levels. Another gene *CUBN* including suggestive SNPs rs12414709 and rs17345993 has been linked to HDL-C metabolism (54). Cubilin (*CUBN*) encodes high affinity HDL-C and lipid-poor apoA-I endocytosis receptor, which participate in the renal clearance of filterable apolipoprotein AI/HDL-C (55, 56).

##### LDL-C level

SNPs rs10490120, rs4953640, rs74263479, rs17037869 were included in the *FSHR* gene. Follicle stimulating hormone receptor (*FSHR*) may interact with FSH in human hepatic tissue, which can elevate LDL-C level by blocking the expression of LDLR (57, 58). Another *KCNK9* around the suggestive SNP rs13251143 also has evidence for dyslipidemia. A great deal of studies has shown that *KCNK9* gene is associated with obesity, HDL-C, adiponectin levels and aldosterone production (59–61). All the above factors are related to dyslipidemia (62–64).

As for cell-type enhancers, the relationship between TC level and ovary has been studied. Women with polycystic ovary syndrome had higher TC and LDL-C level, lower HDL-C level (65, 66). Many studies have showed that Estrogen Receptor 1 (*ESR1*) is associated with TC, HDL-C, LDL-C level (67).

##### Imputation

Genotype imputation substantially increases available SNPs for analysis in GWAS. Substantive SNPs introduced by imputation reached the suggestive level associated with cholesterol. SNPs rs17578959 and rs28845526 were located in the *PID1* gene, which served as the regulator of the LDLR-related protein 1 (*LRP1*) function and controlled the processing of postprandial lipoproteins (68). Solute Carrier Family 13 Member 1 (*SLC13A1*) gene can regulate the cholesterol level by participating in transport of glucose and other sugars, bile salts and organic acids, metal ions and amine compounds pathways.

As additional replication, we cross-referenced our post-imputation results with 34,421 East Asians lipids GWAS meta-analysis results (Supplemental Table 10). A list of SNPs could be replicated, especially the SNPs located in the relevant genes, eg. *RGS5, OSBPL10, SRSF8* for TC level, *KAZN, LOC105378657, LOC105373529, LOC105373941, BIN3, LINC02153, LDHAL6CP* for HDL-C level, *VPS13D, IGF2BP2, PSMB7, CA10, NECTIN2, APOE, APOC1, TOMM40* for LDL-C level.

#### Gene-based analysis

##### TC level

Fas Associated Factor 1 (*FAF1*) can inhibit the activation of NF-kB by interfering with the assembly of IêB kinase (IKK) complex and impeding the nuclear translocation of NF-kB RelA in stimulation-dependent manner. NF-kB signaling pathway is closely related to inflammation (69–71). Epidemiology investigations have revealed that inflammation is associated with a risk of dyslipidemia (72, 73). Lamina C et al. found that Kallikrein B1 (*KLKB1*) was associated with apolipoprotein A-IV concentrations in a genome-wide association meta-analysis. Apolipoprotein A-IV acting as a major component of HDL-C and chylomicron particles participates in reverse cholesterol transport (74).

##### HDL-C level

Kallikrein B1 (*KLKB1*) has been studied in a genome-wide association meta-analysis on apolipoprotein A-IV concentrations. Apolipoprotein A-IV acting as a major component of HDL-C and chylomicron particles participates in reverse cholesterol transport (74).

##### LDL-C level

Neurotrophic Receptor Tyrosine Kinase 1 (*NTRK1*) encodes the neurotrophic tyrosine kinase receptor (NTKR) family members, which are related to classical mitogen-activated protein kinases (MAPK) signaling pathways (75). Further, MAP signaling pathways participated in lipid metabolism (76). Syntrophin Beta 2 (*SNTB2*) suggestes a role in ERK and SR-BI level, and sphingomyelin metabolism in obesity and have been proved to affect the activity of ABCA1 by stabilizing ABCA1 protein, which affect the catabolism of LDL-C level (77–79). Fas Associated Factor 1 (*FAF1*) also plays a key role in regulating the LDL-C level (69–71).

#### Pathway enrichment analysis

##### TC level

Pathway analysis revealed several biological processes significantly associated with TC level: zinc transporters, metal ion SLC transporters, immune system, regulation of transport. Other novel pathways especially top 20 may also be interesting potential candidates for future research and validation.

(1) Zinc serves as a catalytic or structural cofactor for many different proteins and participates in the formation and function of enzyme. One study in young Finns (6–18 years) reported that serum Zinc was positively correlated with TC level (80). (2) Six SLC gene families encode proteins which mediate transport of metals. Transition metal ion such as vanadium, iron, can influence the serum lipid and lipoprotein profiles (81, 82). (3) ERKS ARE inactivated, MAPK targets nuclear events mediated by map kinases and ERK MAPK targets participate in the immune system pathway. Immune system is also associated with cholesterol level. Acute phase conditions and immune disorders promote the decrease of HDL-C and change the composition and size of HDL-C (83). (4) Transport of glucose and other sugars bile salts and organic acids metal ions and amine compounds and amino acid transport across the plasma membrane participate in regulation of transport. Several genes (*GAS6, GPLD1, PRKCE, WNK1, ESR1, RAC2, FAF1, FFAR1, RNASEL, GCK, SLC30A8, RARRES2, TLR5, TLR1, ACSL3, ABCA7, Th17)* enriching in pathway of regulation of transport have been clarified the association with cholesterol level.

##### HDL level

Several biological pathways significantly related to HDL-C levels include: cell adhesion molecules CAMs, tight junction, chondroitin sulfate biosynthesis, sulfur metabolism, immune system, regulation of transport.

(1) An increased CAMs expression may be a mechanism that decreases plasma HDL-C level (84). (2) Zonulin, a physiological mediator of tight intercellular junctions reversibly regulates intestinal permeability to reduce the level of HDL-C (85). (3) Animal experiments have shown that chondroitin-6-sulfate can reduce plasma LDL-C (86). (4) Sulfur-containing compounds are generally associated with an unfavorable lipid profile (87). (5) IL-6 signaling, signaling by ILS, IL6 pathway, natural killer cell mediated cytotoxicity, leukocyte transendothelial migration constitute the immune system to regulate cholesterol levels. (6) Bile salt and organic anion SLC transporters, transport of glucose and other sugars bile salts and organic acids metal ions and amine compounds participate in regulation of transport.

##### LDL level

The pathways associated with LDL-C were: immune system, metabolism of amino acids and derivatives, ERBB signaling pathway, HIV infection.

(1) FC epsilon RI signaling pathway, NFAT pathway, IL1R pathway constitute the immune system to regulate cholesterol levels. (2) Metabolism of amino acids and derivatives participates in the formation and function of enzyme to regulate cholesterol level. (3) ERBB4 activates sterol regulatory element binding protein-2 (SREBP-2) to enhance LDL-C uptake and cholesterol biosynthesis (88). (4) A cross-sectional epidemiological study in China showed that mean LDL-C was lower in HIV-positive than HIV-negative subjects (89).

#### Strengths and limitations

There are two advantages in this study. First, our results were based on the twin data of TC, HDL-C, LDL-C level. Associated individuals, such as twin pairs, will confer increased power in genetic association analysis due to their genetic association (21). And the power of our heritability analysis was above 90%, which suggested that the heritability findings were credible. Second, compared with most GWAS results from European populations, we sampled GWAS from the Qingdao twin population. Our GWAS results of TC, HDL-C, LDL-C level in East Asian population provided a basis for future molecular biology investigations.

This study has two limitations that are noteworthy. First, our study had a relatively small sample size due to the challenges of recruiting and identifying qualified twin pairs. So it should be noted that the power of our analysis might not be sufficient to detect association between SNPs, pathways and cholesterol levels, and our results needed to be furtherly confirmed. Second, our study provided a lot of suggestive results, but no statistically significant results. However, many variants and genes had been confirmed, other novel variants and genes may also be interesting potential candidates for future research and validation.

## Conclusions

In brief, we have verified genetic impact on TC, HDL-C, LDL-C variation through twin modeling. The promising genetic regions for TC, HDL-C, LDL-C level were on chromosome 11 around rs7107698, chromosome 5 around rs12518218, chromosome 2 around rs10490120, respectively. There are 8, 14, 9 SNPs exceeding the threshold for suggestive significance level for TC, HDL-C, LDL-C level, respectively. In the gene-based analysis, the number of genes that was nominally associated with TC, HDL-C, LDL-C level was 1,038, 1,033, and 1,090, respectively. Although our findings need to be replicated and validated, the data reported here could represent a useful reference for GWAS results of TC, HDL-C, LDL-C level in East Asian population and provide a basis for future molecular biology investigations.

## Data availability

The SNPs datasets of this study have been deposited in the European Variation Archive (EVA) (Accession No. PRJEB23749).

## Author contributions

HL and DZ designed the study. HD and CX collected samples and phenotypes. HL and CZ assisted in sample data and sequencing data management. HL and WW analyzed the sequencing data and interpreted the analysis results. HL and DZ drafted the manuscript, XT and HD participated in the discussion, and WW, CX, and CZ revised it. All the authors read the manuscript and agreed to publish. All the authors agreed to be responsible for all aspects of the work.

### Conflict of interest statement

The authors declare that the research was conducted in the absence of any commercial or financial relationships that could be construed as a potential conflict of interest.

## Footnotes

^1^http://www.vcu.edu/mx

^2^http://archive.broadinstitute.org/mammals/haploreg/haploreg.php

^3^https://vegas2.qimrberghofer.edu.au/

^4^http://software.broadinstitute.org/gsea/msigdb

^5^https://blog.nus.edu.sg/agen/summary-statistics/

## Supplementary material

Supplemental Figure 1 Quantile-quantile plot for quality control check and visualizing crude association for genome-wide association study of TC level. The x-axis shows the –log10 of expected *P*-values of association from chi-square distribution and the y-axis shows the –log10 of *P*-values from the observed chi-square distribution. The black dots represent the observed data, and the red line is the expectation under the null hypothesis of no association.

Supplemental Figure 2 Manhattan plot for genome-wide association study of TC level. The x-axis shows the numbers of autosomes and the X chromosome, and the y-axis shows the –log10 of *P*-values for statistical significance. The dots represent the SNPs. None of the SNPs reached the genome-wide significance level (*P* < 5 × 10^−8^).

Supplemental Figure 3 Regional association plot showing signal around chromosomal loci of 11p15.4 for genome-wide association study of TC level.

Supplemental Figure 4 Quantile-quantile plot for quality control check and visualizing crude association for genome-wide association study of LDL-C level. The x-axis shows the –log10 of expected *P*-values of association from chi-square distribution and the y-axis shows the –log10 of *P*-values from the observed chi-square distribution. The black dots represent the observed data, and the red line is the expectation under the null hypothesis of no association.

Supplemental Figure 5 Manhattan plot for genome-wide association study of LDL-C level. The x-axis shows the numbers of autosomes and the X chromosome, and the y-axis shows the –log10 of *P*-values for statistical significance. The dots represent the SNPs. None of the SNPs reached the genome-wide significance level (*P* < 5 × 10^−8^).

Supplemental Figure 6 Regional association plot showing signal around chromosomal loci of 2p16.3 for genome-wide association study of LDL-C level.

Supplemental Figure 7 Quantile-quantile plot for PASCAL pathways results of TC level. The x-axis shows the -log10 of expected *P*-values of association from chi-square distribution and the y-axis shows the –log10 of *P*-values from the observed chi-square distribution. The black dots represent the observed data, and the red line is the expectation under the null hypothesis of no association.

Supplemental Figure 8 Quantile-quantile plot for PASCAL pathway results of HDL-C level. The x-axis shows the –log10 of expected *P*-values of association from chi-square distribution and the y-axis shows the –log10 of *P*-values from the observed chi-square distribution. The black dots represent the observed data, and the red line is the expectation under the null hypothesis of no association.

Supplemental Figure 9 Quantile-quantile plot for PASCAL pathway results of LDL-C level. The x-axis shows the –log10 of expected *P*-values of association from chi-square distribution and the y-axis shows the –log10 of *P*-values from the observed chi-square distribution. The black dots represent the observed data, and the red line is the expectation under the null hypothesis of no association.

Supplemental Table 1 Descriptive statistics for subjects in all sample and GWAS sample.

Supplemental Table 2 Descriptive statistics for subjects in all sample and GWAS sample phenotypic correlation coefficients (95% CI) with covariates' effects in MZ and DZ twin pairs.

Supplemental Table 3 The summary of SNPs with *P* < 1 × 10^−5^ for association with TC in typed GWAS data.

Supplemental Table 4 Query SNP enhancer summary for TC level in typed GWAS data.

Supplemental Table 5 The summary of SNPs with *P* < 1 × 10^−5^ for association with LDL-C in typed GWAS data.

Supplemental Table 6 Query SNP enhancer summary for LDL-C level in typed GWAS data.

Supplemental Table 7 The summary of SNPs with *P* < 1 × 10^−5^ for association with TC in GWAS imputation.

Supplemental Table 8 The summary of SNPs with *P* < 1 × 10^−5^ for association with HDL-C in GWAS imputation.

Supplemental Table 9 The summary of SNPs with *P* < 1 × 10^−5^ for association with LDL-C in GWAS imputation.

Supplemental Table 10 The comparison between our imputation results of genome-wide association study and other East Asian studies.

Supplemental Table 11 The top 20 genes from VEGAS2 gene-based analysis showing the strongest association with TC level in typed GWAS data.

Supplemental Table 12 The top 20 genes from VEGAS2 gene-based analysis showing the strongest association with LDL-C level in typed GWAS data.

Supplemental Table 13 Common genes among TC, HDL-C and LDL-C level (*P* < 0.05).

Supplemental Table 14 The top 20 pathway results-KEGG, Reactome, and Biocarta (emp-*P* < 0.05) using PASCAL program for TC level in typed GWAS data.

Supplemental Table 15 The top 20 pathway results-KEGG, Reactome, and Biocarta (emp-*P* < 0.05) using PASCAL program for LDL-C level in typed GWAS data.

## Abbreviations

DZ: dizygotic
GWAS: genome-wide association study
MZ: monozygotic
TC: total cholesterol
VEGAS2: Versatile Gene-based Association Study-2.
