# Supplementary figures and images for: Heritability and Genome-Wide Association Study of Plasma Cholesterol in Chinese Adult Twins
Source: Front Endocrinol (Lausanne). 2018 Nov 15;9:677. doi: 10.3389/fendo.2018.00677 (PMC6249314; doi:10.3389/fendo.2018.00677)

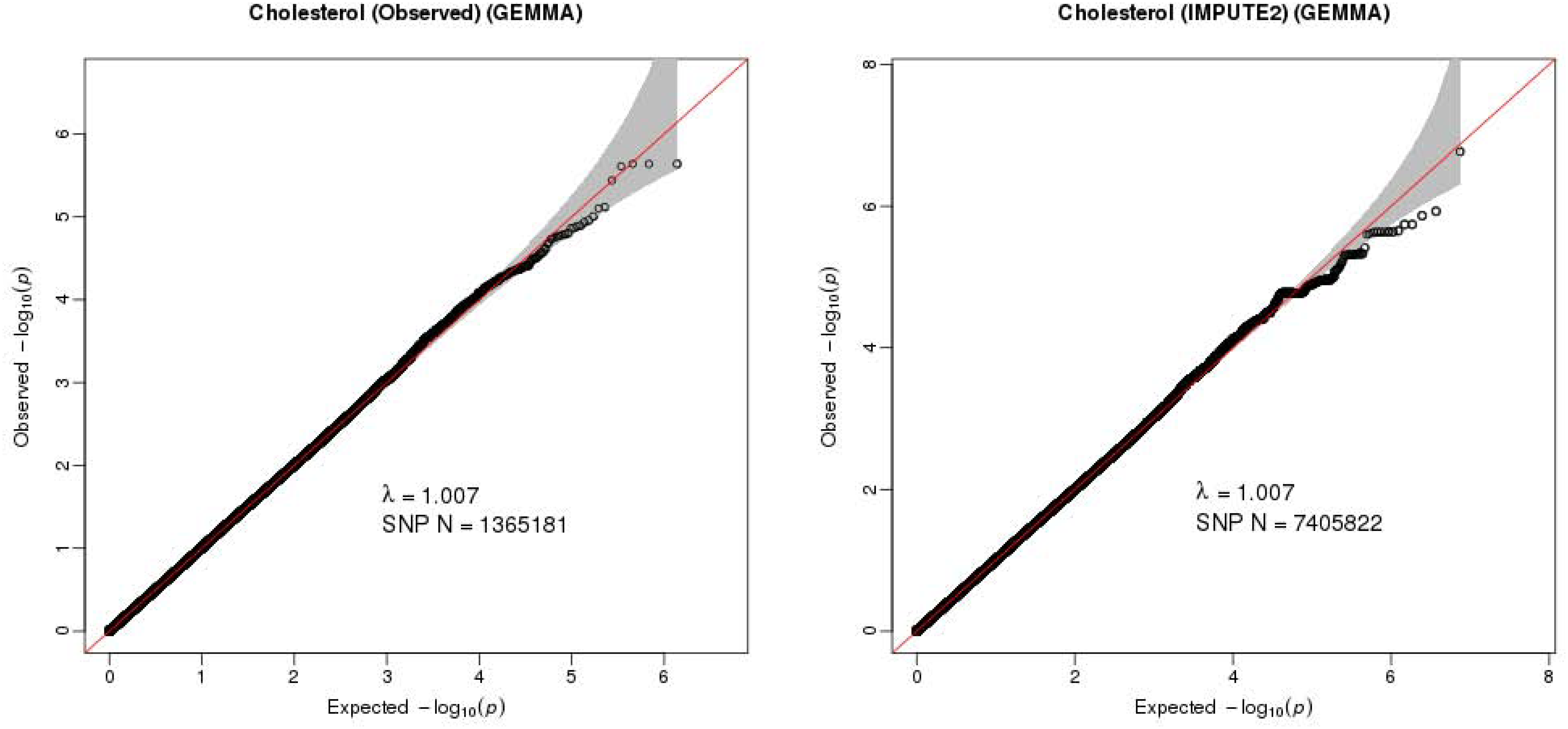

Supplement: Supplemental Figure 1 — Quantile-quantile plot for quality control check and visualizing crude association for genome-wide association study of TC level. The x-axis shows the –log10 of expected P-values of association from chi-square distribution and the y-axis shows the –log10 of P-values from the observed chi-square distribution. The black dots represent the observed data, and the red line is the expectation under the null hypothesis of no association. [file Image_1.TIF]

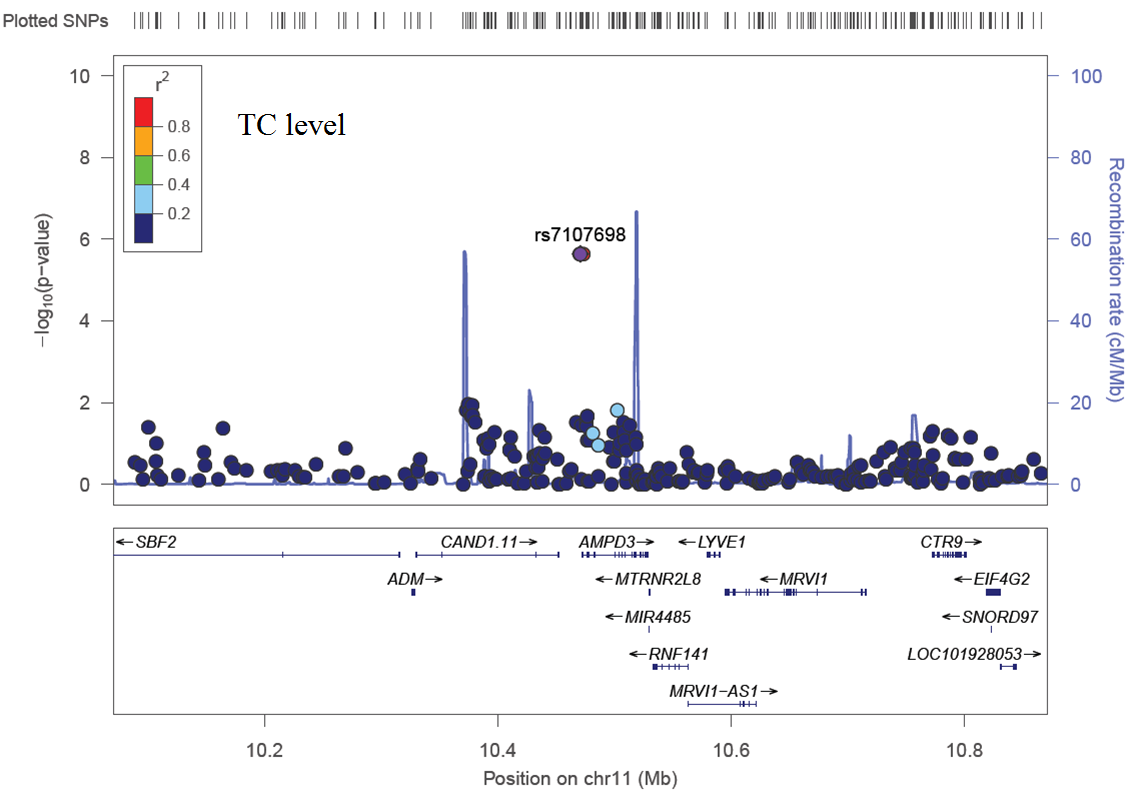

Supplement: Supplemental Figure 3 — Regional association plot showing signal around chromosomal loci of 11p15.4 for genome-wide association study of TC level. [file Image_3.TIF]

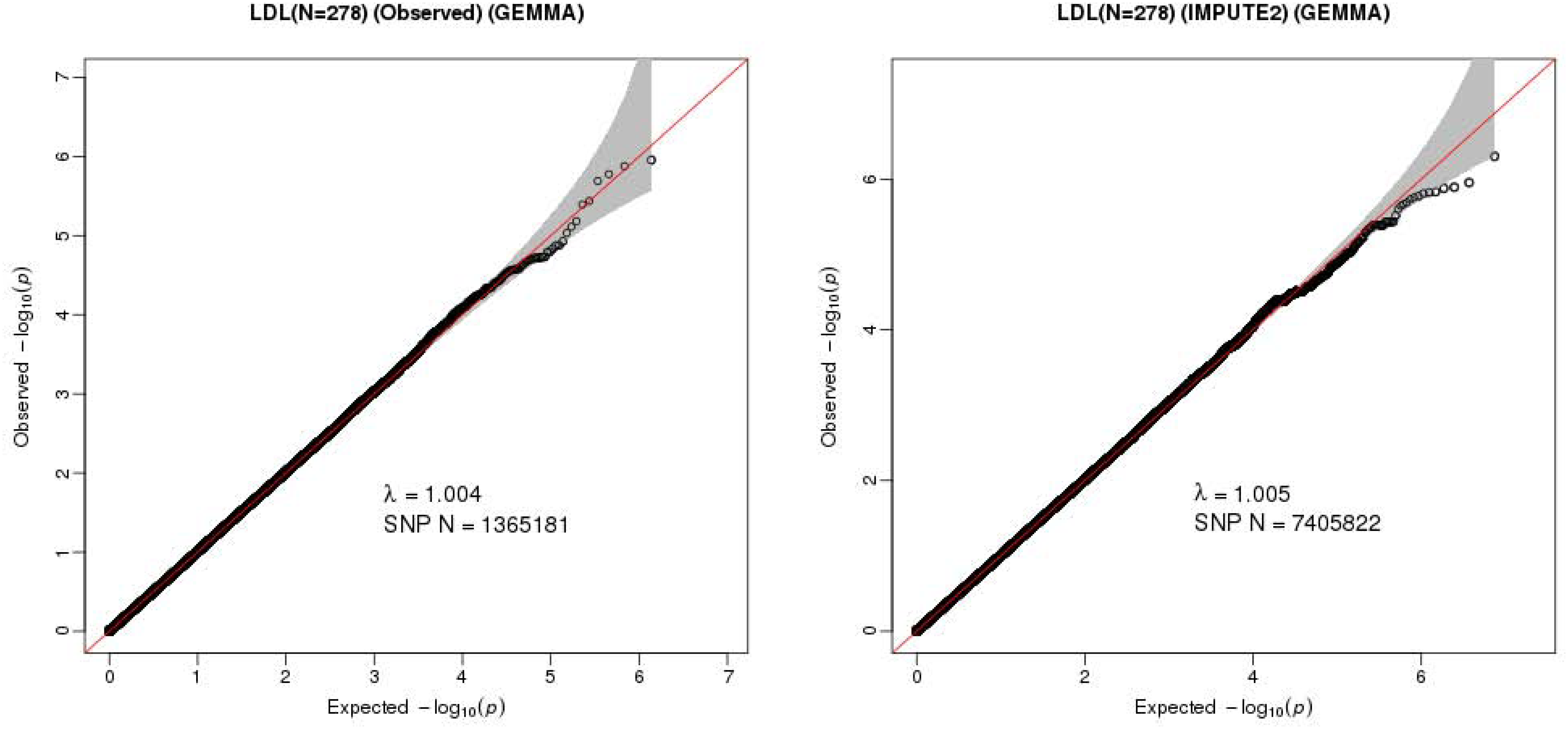

Supplement: Supplemental Figure 4 — Quantile-quantile plot for quality control check and visualizing crude association for genome-wide association study of LDL-C level. The x-axis shows the –log10 of expected P-values of association from chi-square distribution and the y-axis shows the –log10 of P-values from the observed chi-square distribution. The black dots represent the observed data, and the red line is the expectation under the null hypothesis of no association. [file Image_4.TIF]

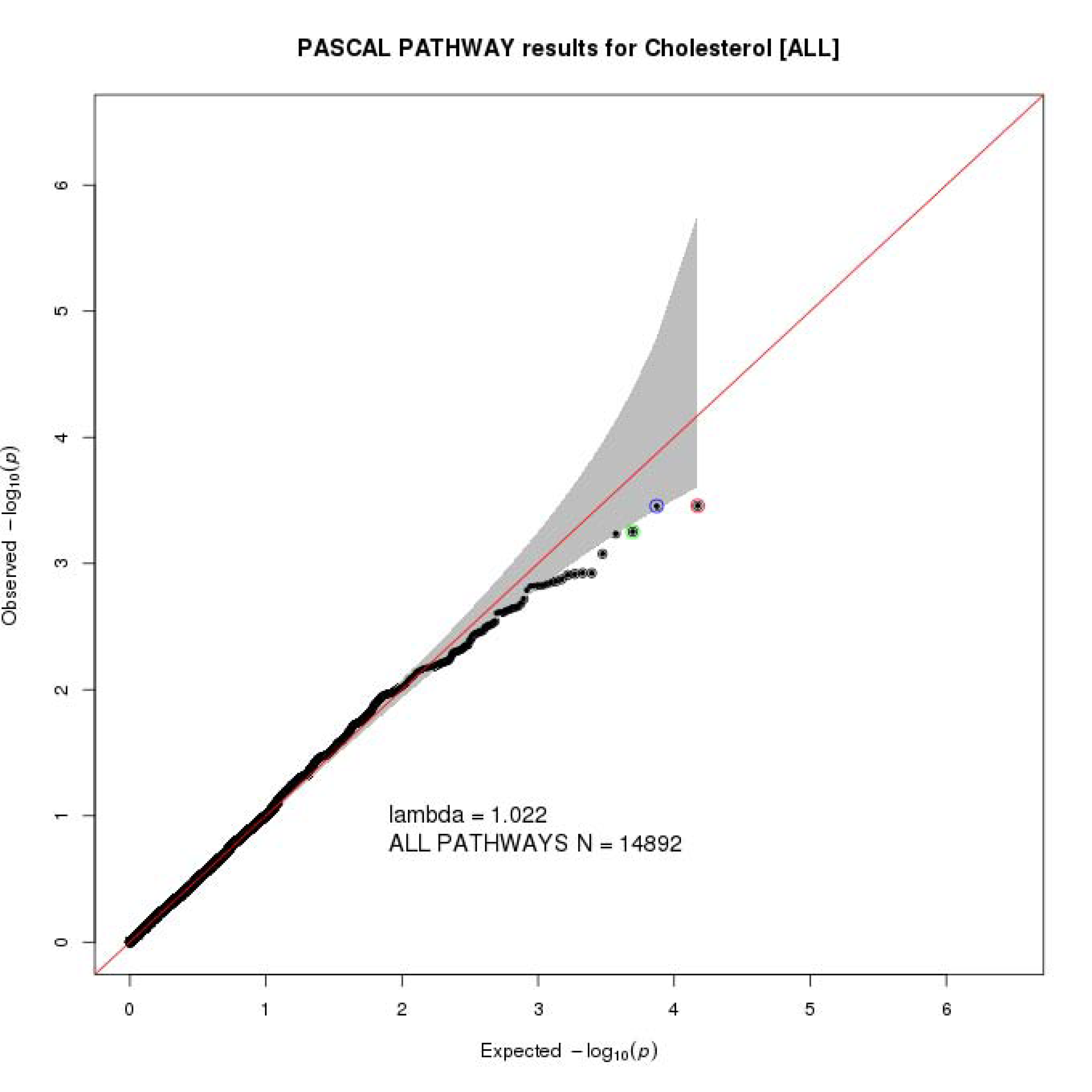

Supplement: Supplemental Figure 7 — Quantile-quantile plot for PASCAL pathways results of TC level. The x-axis shows the -log10 of expected P-values of association from chi-square distribution and the y-axis shows the –log10 of P-values from the observed chi-square distribution. The black dots represent the observed data, and the red line is the expectation under the null hypothesis of no association. [file Image_7.TIF]

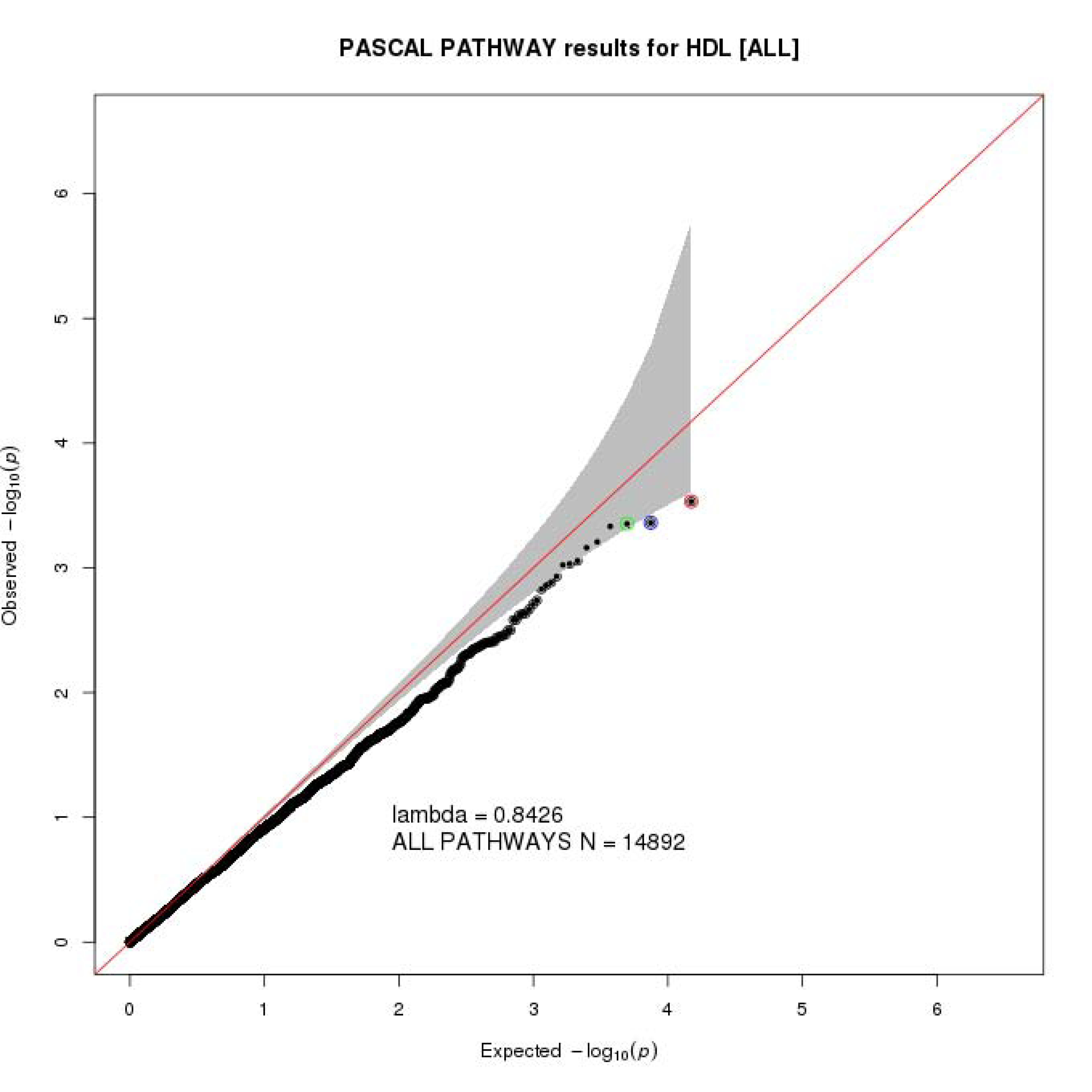

Supplement: Supplemental Figure 8 — Quantile-quantile plot for PASCAL pathway results of HDL-C level. The x-axis shows the –log10 of expected P-values of association from chi-square distribution and the y-axis shows the –log10 of P-values from the observed chi-square distribution. The black dots represent the observed data, and the red line is the expectation under the null hypothesis of no association. [file Image_8.TIF]

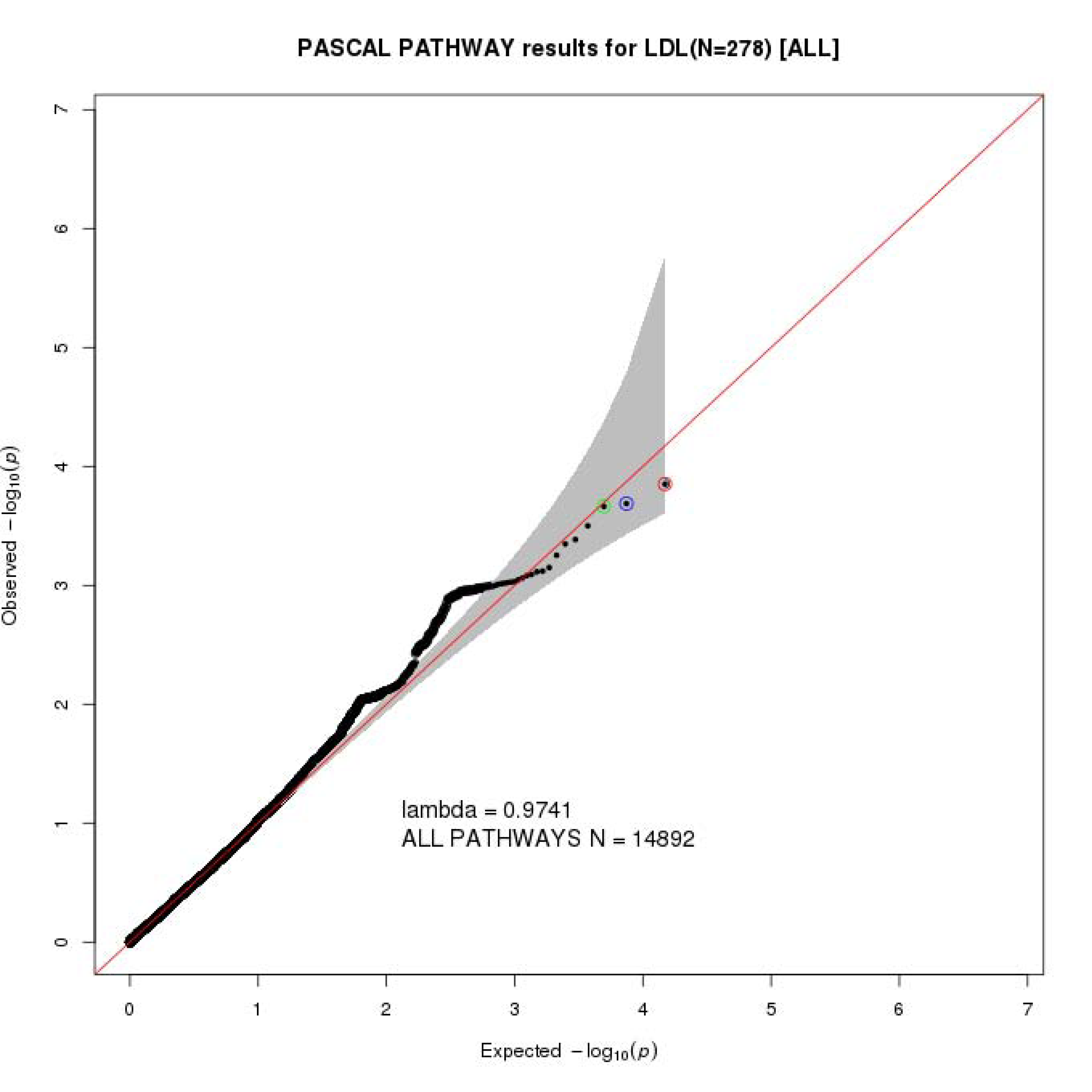

Supplement: Supplemental Figure 9 — Quantile-quantile plot for PASCAL pathway results of LDL-C level. The x-axis shows the –log10 of expected P-values of association from chi-square distribution and the y-axis shows the –log10 of P-values from the observed chi-square distribution. The black dots represent the observed data, and the red line is the expectation under the null hypothesis of no association. [file Image_9.TIF]
