# Supplementary material for: Heritability and Genome-Wide Association Study of Plasma Cholesterol in Chinese Adult Twins
Source: Front Endocrinol (Lausanne). 2018 Nov 15;9:677. doi: 10.3389/fendo.2018.00677 (PMC6249314; doi:10.3389/fendo.2018.00677)
Supplement: Supplemental Table 1 — Descriptive statistics for subjects in all sample and GWAS sample. [file Table_1.DOCX]

|  | Variable | Male | | Female | | All | |
| --- | --- | --- | --- | --- | --- | --- | --- |
|  |  | N | Mean ± SD | N | Mean ± SD | N | Mean ± SD |
|  | Age (years)^*^ | 371 | 52.3 ± 8.7 | 393 | 50.9 ± 6.7 | 764 | 51.6 ± 7.7 |
| All | TC (umol/L)^*^ | 371 | 4.8 ± 1.2 | 393 | 5.1 ± 1.1 | 764 | 4.9 ± 1.2 |
| sample | HDL-C(umol/L)^*^ | 371 | 1.3 ± 0.4 | 393 | 1.7 ± 0.6 | 764 | 1.5 ± 0.5 |
|  | LDL-C(umol/L)^*^ | 371 | 2.8 ± 0.9 | 393 | 2.8 ± 0.9 | 764 | 2.8 ± 0.9 |
|  | Age (years)^*^ | 141 | 51.0 ± 7.0 | 137 | 51.2 ± 7.0 | 278 | 51.1 ± 7.0 |
| GWAS | TC(umol/L)^*^ | 141 | 5.0 ± 1.3 | 137 | 5.1 ± 1.1 | 278 | 5.0 ± 1.2 |
| sample | HDL-C(umol/L)^*^ | 141 | 1.3 ± 0.4 | 137 | 1.7 ± 0.6 | 278 | 1.5 ± 0.5 |
|  | LDL-C(umol/L)^*^ | 141 | 2.9± 1.1 | 137 | 2.8 ± 0.9 | 278 | 2.9 ± 1.0 |

**Supplemental Table 1** Descriptive statistics for subjects in all sample and GWAS sample

**Note**: GWAS, genome-wide association study; HDL-C, high density lipoprotein cholesterol; LDL-C, low density lipoprotein cholesterol; Mean ± SD, mean value ±standard deviation; N, number of subjects; TC, total cholesterol; *, All indicators were skewed and converted into normal distribution by Blom’s formula.
