# Supplementary material for: Heritability and Genome-Wide Association Study of Plasma Cholesterol in Chinese Adult Twins
Source: Front Endocrinol (Lausanne). 2018 Nov 15;9:677. doi: 10.3389/fendo.2018.00677 (PMC6249314; doi:10.3389/fendo.2018.00677)
Supplement: Supplemental Table 2 — Descriptive statistics for subjects in all sample and GWAS sample phenotypic correlation coefficients (95% CI) with covariates' effects in MZ and DZ twin pairs. [file Table_2.DOCX]

| Variable | Model | Twin correlation | |  | |  | Covariate effects | | |  |
| --- | --- | --- | --- | --- | --- | --- | --- | --- | --- | --- |
|  |  | MZ (95% CI) | | DZ (95% CI) | | | -2LL | df | χ^2^ | *P* |
| TC | Base | 0.61 | (0.52-0.68) | 0.35 | (0.21-0.47) | | 1996.90 | 757 |  |  |
|  | No sex | 0.61 | (0.53-0.68) | 0.37 | (0.23-0.48) | | 2002.60 | 758 | 5.64 | 1.76E-02 |
|  | No age | 0.62 | (0.54-0.69) | 0.36 | (0.23-0.48) | | 2009.10 | 758 | 12.17 | 4.85E-04 |
|  | No education | 0.63 | (0.55-0.70) | 0.35 | (0.21-0.47) | | 2005.90 | 758 | 8.99 | 2.72E-03 |
| HDL-C | Base | 0.74 | (0.68-0.79) | 0.61 | (0.50-0.70) | | 1763.60 | 757 |  |  |
|  | No sex | 0.77 | (0.71-0.81) | 0.61 | (0.50-0.70) | | 1818.70 | 758 | 55.09 | 1.15E-13 |
|  | No age | 0.75 | (0.70-0.80) | 0.62 | (0.52-0.70) | | 1771.90 | 758 | 8.33 | 3.90E-03 |
|  | No education | 0.77 | (0.72-0.81) | 0.62 | (0.52-0.71) | | 1784.00 | 758 | 20.45 | 6.13E-06 |
| LDL-C | Base | 0.65 | (0.57-0.72) | 0.35 | (0.21-0.46) | | 2028.10 | 760 |  |  |
|  | No sex | 0.65 | (0.57-0.72) | 0.34 | (0.21-0.46) | | 2028.30 | 761 | 0.14 | 7.04E-01 |
|  | No age | 0.66 | (0.58-0.72) | 0.35 | (0.22-0.47) | | 2033.70 | 761 | 5.58 | 1.81E-02 |
|  | No education | 0.65 | (0.57-0.72) | 0.34 | (0.21-0.46) | | 2028.30 | 761 | 0.15 | 6.96E-01 |

**Supplemental Table 2** Descriptive statistics for subjects in all sample and GWAS sample phenotypic correlation coefficients (95% CI) with covariates' effects in MZ and DZ twin pairs

**Note**: 95%CI, 95% confidence intervals; -2LL, -2 Log Likelihood; df, degree of freedom; χ^2^, difference of χ^2^ value; *P*, χ^2^ test in model fitting; DZ, dizygotic; HDL-C, high density lipoprotein cholesterol; LDL-C, low density lipoprotein cholesterol; MZ, monozygotic; TC, total cholesterol;
