# Supplementary material for: Heritability and Genome-Wide Association Study of Plasma Cholesterol in Chinese Adult Twins
Source: Front Endocrinol (Lausanne). 2018 Nov 15;9:677. doi: 10.3389/fendo.2018.00677 (PMC6249314; doi:10.3389/fendo.2018.00677)
Supplement: Supplemental Table 3 — The summary of SNPs with P < 1 × 10^−5^ for association with TC in typed GWAS data. [file Table_3.DOCX]

**Supplemental Table 3** The summary of SNPs with P-value <1×10^-5^ for association with TC in typed GWAS data

| SNP | Chr band | CHR | BP | *P*-value | Closest genes or genes | Official full name |  |
| --- | --- | --- | --- | --- | --- | --- | --- |
| **rs7107698** | 11p15.4 | 11 | 10,470,744 | 2.29E-06 | *AMPD3* | Adenosine monophosphate deaminase 3 | |
| **rs4909928** | 11p15.4 | 11 | 10,470,275 | 2.29E-06 | *AMPD3* | Adenosine monophosphate deaminase 3 | |
| **rs12184411** | 11p15.4 | 11 | 10,473,170 | 2.29E-06 | *AMPD3* | Adenosine monophosphate deaminase 3 | |
| **kgp6520322**  (rs10840418) | 11p15.4 | 11 | 10,467,636 | 2.45E-06 | *AMPD3* | Adenosine monophosphate deaminase 3 | |
| rs3263 | 17p13.3 | 17 | 908,795 | 3.62E-06 | *ABR* | Active BCR-related | |
| rs310892 | 5p14.3,5p14.2 | 5 | 23,195,394 | 7.59E-06 | *LOC105374687* | Uncharacterized LOC105374687 | |
| rs13129710 | 4q28.3 | 4 | 138,229,547 | 7.88E-06 | *LOC105377441* | Long intergenic non-protein coding RNA 2511 | |
| rs1425971 | 4q28.3 | 4 | 138,228,499 | 9.81E-06 | *LOC105377441* | Long intergenic non-protein coding RNA 2511 | |

**Note**: kgp, 1000 Genomes Project; CHR, chromosome; The content discussed in detail were in bold.
