# Supplementary material for: Heritability and Genome-Wide Association Study of Plasma Cholesterol in Chinese Adult Twins
Source: Front Endocrinol (Lausanne). 2018 Nov 15;9:677. doi: 10.3389/fendo.2018.00677 (PMC6249314; doi:10.3389/fendo.2018.00677)
Supplement: Supplemental Table 4 — Query SNP enhancer summary for TC level in typed GWAS data. [file Table_4.DOCX]

**Supplemental Table 4** Query SNP enhancer summary for TC level in typed GWAS data

| **Cell** | **Observed** | **Expected** | **Expected** | **Binomial *P*** | **Binomial *P*** |
| --- | --- | --- | --- | --- | --- |
|  |  | **(all SNPs)** | **(GWAS SNPs)** | **(all SNPs)** | **(GWAS SNPs)** |
| E017 LNG.IMR90 (IMR90 fetal lung fibroblasts Cell Line) | 2 | 0.3 | 0.6 | **0.034856** | 0.106511 |
| E002 ESC.WA7 (ES-WA7 Cells) | 1 | 0.1 | 0.2 | 0.092143 | 0.155865 |
| E008 ESC.H9 (H9 Cells) | 0 | 0.1 | 0.2 | 1 | 1 |
| E001 ESC.I3 (ES-I3 Cells) | 1 | 0.3 | 0.4 | 0.239657 | 0.358083 |
| E015 ESC.HUES6 (HUES6 Cells) | 0 | 0.3 | 0.4 | 1 | 1 |
| E014 ESC.HUES48 (HUES48 Cells) | 0 | 0.3 | 0.4 | 1 | 1 |
| E016 ESC.HUES64 (HUES64 Cells) | 0 | 0.2 | 0.4 | 1 | 1 |
| E003 ESC.H1 (H1 Cells) | 0 | 0.2 | 0.4 | 1 | 1 |
| E024 ESC.4STAR (ES-UCSF4 Cells) | 0 | 0.3 | 0.5 | 1 | 1 |
| E020 IPSC.20B (iPS-20b Cells) | 0 | 0.2 | 0.3 | 1 | 1 |
| E019 IPSC.18 (iPS-18 Cells) | 0 | 0.3 | 0.4 | 1 | 1 |
| E018 IPSC.15b (iPS-15b Cells) | 0 | 0.2 | 0.4 | 1 | 1 |
| E021 IPSC.DF.6.9 (iPS DF 6.9 Cells) | 0 | 0.1 | 0.2 | 1 | 1 |
| E022 IPSC.DF.19.11 (iPS DF 19.11 Cells) | 0 | 0.3 | 0.5 | 1 | 1 |
| E007 ESDR.H1.NEUR.PROG (H1 Derived Neuronal Progenitor Cultured Cells) | 0 | 0.1 | 0.3 | 1 | 1 |
| E009 ESDR.H9.NEUR.PROG (H9 Derived Neuronal Progenitor Cultured Cells) | 0 | 0.2 | 0.3 | 1 | 1 |
| E010 ESDR.H9.NEUR (H9 Derived Neuron Cultured Cells) | 1 | 0.2 | 0.4 | 0.220575 | 0.343748 |
| E013 ESDR.CD56.MESO (hESC Derived CD56+ Mesoderm Cultured Cells) | 0 | 0.2 | 0.4 | 1 | 1 |
| E012 ESDR.CD56.ECTO (hESC Derived CD56+ Ectoderm Cultured Cells) | 0 | 0.2 | 0.4 | 1 | 1 |
| E011 ESDR.CD184.ENDO (hESC Derived CD184+ Endoderm Cultured Cells) | 0 | 0.2 | 0.4 | 1 | 1 |
| E004 ESDR.H1.BMP4.MESO (H1 BMP4 Derived Mesendoderm Cultured Cells) | 0 | 0.1 | 0.2 | 1 | 1 |
| E005 ESDR.H1.BMP4.TROP (H1 BMP4 Derived Trophoblast Cultured Cells) | 0 | 0.3 | 0.5 | 1 | 1 |
| E006 ESDR.H1.MSC (H1 Derived Mesenchymal Stem Cells) | 1 | 0.3 | 0.5 | 0.242712 | 0.402835 |
| E062 BLD.PER.MONUC.PC (Primary mononuclear cells from peripheral blood) | 0 | 0.1 | 0.2 | 1 | 1 |
| E034 BLD.CD3.PPC (Primary T cells from peripheral blood) | 0 | 0.2 | 0.4 | 1 | 1 |
| E045 BLD.CD4.CD25I.CD127.TMEMPC (Primary T cells effector/memory enriched from peripheral blood) | 0 | 0.1 | 0.2 | 1 | 1 |
| E033 BLD.CD3.CPC (Primary T cells from cord blood) | 0 | 0.2 | 0.3 | 1 | 1 |
| E044 BLD.CD4.CD25.CD127M.TREGPC (Primary T regulatory cells from peripheral blood) | 0 | 0.1 | 0.3 | 1 | 1 |
| E043 BLD.CD4.CD25M.TPC (Primary T helper cells from peripheral blood) | 0 | 0.2 | 0.4 | 1 | 1 |
| E039 BLD.CD4.CD25M.CD45RA.NPC (Primary T helper naive cells from peripheral blood) | 0 | 0.2 | 0.3 | 1 | 1 |
| E041 BLD.CD4.CD25M.IL17M.PL.TPC (Primary T helper cells PMA-I stimulated) | 0 | 0.2 | 0.4 | 1 | 1 |
| E042 BLD.CD4.CD25M.IL17P.PL.TPC (Primary T helper 17 cells PMA-I stimulated) | 0 | 0.2 | 0.3 | 1 | 1 |
| E040 BLD.CD4.CD25M.CD45RO.MPC (Primary T helper memory cells from peripheral blood 1) | 0 | 0.2 | 0.3 | 1 | 1 |
| E037 BLD.CD4.MPC (Primary T helper memory cells from peripheral blood 2) | 0 | 0.2 | 0.4 | 1 | 1 |
| E048 BLD.CD8.MPC (Primary T CD8+ memory cells from peripheral blood) | 0 | 0.2 | 0.3 | 1 | 1 |
| E038 BLD.CD4.NPC (Primary T helper naive cells from peripheral blood) | 0 | 0.2 | 0.3 | 1 | 1 |
| E047 BLD.CD8.NPC (Primary T CD8+ naive cells from peripheral blood) | 0 | 0.2 | 0.4 | 1 | 1 |
| E029 BLD.CD14.PC (Primary monocytes from peripheral blood) | 0 | 0.3 | 0.5 | 1 | 1 |
| E031 BLD.CD19.CPC (Primary B cells from cord blood) | 0 | 0.2 | 0.3 | 1 | 1 |
| E035 BLD.CD34.PC (Primary hematopoietic stem cells) | 0 | 0.2 | 0.4 | 1 | 1 |
| E051 BLD.MOB.CD34.PC.M (Primary hematopoietic stem cells G-CSF-mobilized Male) | 0 | 0.3 | 0.5 | 1 | 1 |
| E050 BLD.MOB.CD34.PC.F (Primary hematopoietic stem cells G-CSF-mobilized Female) | 0 | 0.3 | 0.5 | 1 | 1 |
| E036 BLD.CD34.CC (Primary hematopoietic stem cells short term culture) | 1 | 0.3 | 0.5 | 0.237436 | 0.396971 |
| E032 BLD.CD19.PPC (Primary B cells from peripheral blood) | 0 | 0.3 | 0.5 | 1 | 1 |
| E046 BLD.CD56.PC (Primary Natural Killer cells from peripheral blood) | 0 | 0.2 | 0.4 | 1 | 1 |
| E030 BLD.CD15.PC (Primary neutrophils from peripheral blood) | 2 | 0.2 | 0.3 | **0.016566** | **0.042974** |
| E026 STRM.MRW.MSC (Bone Marrow Derived Cultured Mesenchymal Stem Cells) | 1 | 0.2 | 0.4 | 0.221691 | 0.36427 |
| E049 STRM.CHON.MRW.DR.MSC (Mesenchymal Stem Cell Derived Chondrocyte Cultured Cells) | 1 | 0.3 | 0.5 | 0.257652 | 0.434619 |
| E025 FAT.ADIP.DR.MSC (Adipose Derived Mesenchymal Stem Cell Cultured Cells) | 0 | 0.4 | 0.7 | 1 | 1 |
| E023 FAT.MSC.DR.ADIP (Mesenchymal Stem Cell Derived Adipocyte Cultured Cells) | 0 | 0.3 | 0.5 | 1 | 1 |
| E052 MUS.SAT (Muscle Satellite Cultured Cells) | 2 | 0.3 | 0.5 | **0.028788** | 0.07992 |
| E055 SKIN.PEN.FRSK.FIB.01 (Foreskin Fibroblast Primary Cells skin01) | 1 | 0.3 | 0.5 | 0.264228 | 0.433822 |
| E056 SKIN.PEN.FRSK.FIB.02 (Foreskin Fibroblast Primary Cells skin02) | 0 | 0.2 | 0.4 | 1 | 1 |
| E059 SKIN.PEN.FRSK.MEL.01 (Foreskin Melanocyte Primary Cells skin01) | 1 | 0.2 | 0.3 | 0.158435 | 0.283627 |
| E061 SKIN.PEN.FRSK.MEL.03 (Foreskin Melanocyte Primary Cells skin03) | 0 | 0.3 | 0.5 | 1 | 1 |
| E057 SKIN.PEN.FRSK.KER.02 (Foreskin Keratinocyte Primary Cells skin02) | 0 | 0.3 | 0.5 | 1 | 1 |
| E058 SKIN.PEN.FRSK.KER.03 (Foreskin Keratinocyte Primary Cells skin03) | 0 | 0.3 | 0.5 | 1 | 1 |
| E028 BRST.HMEC.35 (Breast variant Human Mammary Epithelial Cells (vHMEC)) | 1 | 0.3 | 0.5 | 0.26437 | 0.426611 |
| E027 BRST.MYO (Breast Myoepithelial Primary Cells) | 0 | 0.4 | 0.7 | 1 | 1 |
| E054 BRN.GANGEM.DR.NRSPHR (Ganglion Eminence derived primary cultured neurospheres) | 0 | 0.2 | 0.3 | 1 | 1 |
| E053 BRN.CRTX.DR.NRSPHR (Cortex derived primary cultured neurospheres) | 1 | 0.3 | 0.4 | 0.239444 | 0.347357 |
| E112 THYM (Thymus) | 0 | 0.1 | 0.2 | 1 | 1 |
| E093 THYM.FET (Fetal Thymus) | 0 | 0.3 | 0.5 | 1 | 1 |
| E071 BRN.HIPP.MID (Brain Hippocampus Middle) | 0 | 0.2 | 0.4 | 1 | 1 |
| E074 BRN.SUB.NIG (Brain Substantia Nigra) | 0 | 0.2 | 0.4 | 1 | 1 |
| E068 BRN.ANT.CAUD (Brain Anterior Caudate) | 0 | 0.2 | 0.4 | 1 | 1 |
| E069 BRN.CING.GYR (Brain Cingulate Gyrus) | 0 | 0.2 | 0.4 | 1 | 1 |
| E072 BRN.INF.TMP (Brain Inferior Temporal Lobe) | 0 | 0.2 | 0.3 | 1 | 1 |
| E067 BRN.ANG.GYR (Brain Angular Gyrus) | 0 | 0.2 | 0.3 | 1 | 1 |
| E073 BRN.DL.PRFRNTL.CRTX (Brain Dorsolateral Prefrontal Cortex) | 0 | 0.1 | 0.3 | 1 | 1 |
| E070 BRN.GRM.MTRX (Brain Germinal Matrix) | 0 | 0.2 | 0.3 | 1 | 1 |
| E082 BRN.FET.F (Fetal Brain Female) | 1 | 0.1 | 0.2 | 0.115815 | 0.181342 |
| E081 BRN.FET.M (Fetal Brain Male) | 1 | 0.2 | 0.3 | 0.218315 | 0.296207 |
| E063 FAT.ADIP.NUC (Adipose Nuclei) | 1 | 0.3 | 0.5 | 0.243437 | 0.432227 |
| E100 MUS.PSOAS (Psoas Muscle) | 1 | 0.1 | 0.3 | 0.139421 | 0.231361 |
| E108 MUS.SKLT.F (Skeletal Muscle Female) | 0 | 0.3 | 0.5 | 1 | 1 |
| E107 MUS.SKLT.M (Skeletal Muscle Male) | 1 | 0.3 | 0.5 | 0.24583 | 0.407822 |
| E089 MUS.TRNK.FET (Fetal Muscle Trunk) | 1 | 0.3 | 0.5 | 0.264247 | 0.431427 |
| E090 MUS.LEG.FET (Fetal Muscle Leg) | 1 | 0.4 | 0.7 | 0.331608 | 0.541748 |
| E083 HRT.FET (Fetal Heart) | 1 | 0.4 | 0.6 | 0.308984 | 0.467971 |
| E104 HRT.ATR.R (Right Atrium) | 1 | 0.2 | 0.3 | 0.164212 | 0.293321 |
| E095 HRT.VENT.L (Left Ventricle) | 0 | 0.2 | 0.4 | 1 | 1 |
| E105 HRT.VNT.R (Right Ventricle) | 1 | 0.2 | 0.4 | 0.174765 | 0.309542 |
| E065 VAS.AOR (Aorta) | 0 | 0.1 | 0.2 | 1 | 1 |
| E078 GI.DUO.SM.MUS (Duodenum Smooth Muscle) | 0 | 0.1 | 0.3 | 1 | 1 |
| E076 GI.CLN.SM.MUS (Colon Smooth Muscle) | 2 | 0.2 | 0.4 | **0.01837** | 0.055185 |
| E103 GI.RECT.SM.MUS (Rectal Smooth Muscle) | 0 | 0.1 | 0.3 | 1 | 1 |
| E111 GI.STMC.MUS (Stomach Smooth Muscle) | 0 | 0.2 | 0.3 | 1 | 1 |
| E092 GI.STMC.FET (Fetal Stomach) | 1 | 0.3 | 0.5 | 0.226164 | 0.395287 |
| E085 GI.S.INT.FET (Fetal Intestine Small) | 1 | 0.3 | 0.5 | 0.237599 | 0.385095 |
| E084 GI.L.INT.FET (Fetal Intestine Large) | 1 | 0.3 | 0.5 | 0.228408 | 0.379945 |
| E109 GI.S.INT (Small Intestine) | 0 | 0.1 | 0.2 | 1 | 1 |
| E106 GI.CLN.SIG (Sigmoid Colon) | 1 | 0.1 | 0.3 | 0.130884 | 0.253859 |
| E075 GI.CLN.MUC (Colonic Mucosa) | 0 | 0.1 | 0.2 | 1 | 1 |
| E101 GI.RECT.MUC.29 (Rectal Mucosa Donor 29) | 0 | 0.1 | 0.2 | 1 | 1 |
| E102 GI.RECT.MUC.31 (Rectal Mucosa Donor 31) | 0 | 0.2 | 0.3 | 1 | 1 |
| E110 GI.STMC.MUC (Stomach Mucosa) | 1 | 0.2 | 0.4 | 0.221703 | 0.355416 |
| E077 GI.DUO.MUC (Duodenum Mucosa) | 0 | 0.2 | 0.4 | 1 | 1 |
| E079 GI.ESO (Esophagus) | 0 | 0.1 | 0.3 | 1 | 1 |
| E094 GI.STMC.GAST (Gastric) | 0 | 0.1 | 0.3 | 1 | 1 |
| E099 PLCNT.AMN (Placenta Amnion) | 1 | 0.2 | 0.3 | 0.14945 | 0.290425 |
| E086 KID.FET (Fetal Kidney) | 0 | 0.1 | 0.2 | 1 | 1 |
| E088 LNG.FET (Fetal Lung) | 0 | 0.3 | 0.5 | 1 | 1 |
| E097 OVRY (Ovary) | 2 | 0.2 | 0.3 | **0.013446** | **0.040332** |
| E087 PANC.ISLT (Pancreatic Islets) | 0 | 0.1 | 0.1 | 1 | 1 |
| E080 ADRL.GLND.FET (Fetal Adrenal Gland) | 1 | 0.3 | 0.6 | 0.293985 | 0.485108 |
| E091 PLCNT.FET (Placenta) | 1 | 0.3 | 0.6 | 0.279731 | 0.484373 |
| E066 LIV.ADLT (Liver) | 0 | 0.3 | 0.5 | 1 | 1 |
| E098 PANC (Pancreas) | 0 | 0.2 | 0.4 | 1 | 1 |
| E096 LNG (Lung) | 0 | 0.2 | 0.3 | 1 | 1 |
| E113 SPLN (Spleen) | 1 | 0.2 | 0.4 | 0.200321 | 0.366906 |
| E114 LNG.A549.ETOH002.CNCR (A549 EtOH 0.02pct Lung Carcinoma Cell Line) | 0 | 0.2 | 0.4 | 1 | 1 |
| E115 BLD.DND41.CNCR (Dnd41 TCell Leukemia Cell Line) | 1 | 0.2 | 0.2 | 0.153665 | 0.201888 |
| E116 BLD.GM12878 (GM12878 Lymphoblastoid Cells) | 0 | 0.2 | 0.3 | 1 | 1 |
| E117 CRVX.HELAS3.CNCR (HeLa-S3 Cervical Carcinoma Cell Line) | 0 | 0.2 | 0.4 | 1 | 1 |
| E118 LIV.HEPG2.CNCR (HepG2 Hepatocellular Carcinoma Cell Line) | 0 | 0.3 | 0.6 | 1 | 1 |
| E119 BRST.HMEC (HMEC Mammary Epithelial Primary Cells) | 0 | 0.3 | 0.5 | 1 | 1 |
| E120 MUS.HSMM (HSMM Skeletal Muscle Myoblasts Cells) | 1 | 0.2 | 0.4 | 0.202915 | 0.371278 |
| E121 MUS.HSMMT (HSMM cell derived Skeletal Muscle Myotubes Cells) | 2 | 0.2 | 0.4 | **0.019113** | 0.057501 |
| E122 VAS.HUVEC (HUVEC Umbilical Vein Endothelial Primary Cells) | 0 | 0.2 | 0.4 | 1 | 1 |
| E123 BLD.K562.CNCR (K562 Leukemia Cells) | 0 | 0.2 | 0.3 | 1 | 1 |
| E124 BLD.CD14.MONO (Monocytes-CD14+ RO01746 Primary Cells) | 0 | 0.2 | 0.3 | 1 | 1 |
| E125 BRN.NHA (NH-A Astrocytes Primary Cells) | 2 | 0.2 | 0.4 | **0.017348** | 0.055843 |
| E126 SKIN.NHDFAD (NHDF-Ad Adult Dermal Fibroblast Primary Cells) | 3 | 0.3 | 0.5 | **0.002295** | **0.01075** |
| E127 SKIN.NHEK (NHEK-Epidermal Keratinocyte Primary Cells) | 0 | 0.3 | 0.5 | 1 | 1 |
| E128 LNG.NHLF (NHLF Lung Fibroblast Primary Cells) | 2 | 0.2 | 0.4 | **0.015197** | 0.050981 |
| E129 BONE.OSTEO (Osteoblast Primary Cells) | 1 | 0.3 | 0.5 | 0.235716 | 0.374757 |
