# Supplementary material for: Heritability and Genome-Wide Association Study of Plasma Cholesterol in Chinese Adult Twins
Source: Front Endocrinol (Lausanne). 2018 Nov 15;9:677. doi: 10.3389/fendo.2018.00677 (PMC6249314; doi:10.3389/fendo.2018.00677)
Supplement: Supplemental Table 5 — The summary of SNPs with P < 1 × 10^−5^ for association with LDL-C in typed GWAS data. [file Table_5.DOCX]

**Supplemental Table 5** The summary of SNPs with P-value <1×10^-5^ for association with LDL-C in typed GWAS data

| SNP | Chr band | CHR | BP | *P*-value | Closest genes or genes | Official full name | |  |
| --- | --- | --- | --- | --- | --- | --- | --- | --- |
| **rs10490120** | 2p16.3 | 2 | 49,143,829 | 1.11E-06 | *FSHR* | Follicle stimulating hormone receptor | | |
| **rs4953640** | 2p16.3 | 2 | 49,126,802 | 1.32E-06 | *FSHR* | Follicle stimulating hormone receptor | | |
| **rs74263479** | 2p16.3 | 2 | 49,132,194 | 1.67E-06 | *FSHR* | Follicle stimulating hormone receptor | | |
| rs367881 | 1p31.1 | 1 | 82,348,717 | 2.04E-06 | *ADGRL2* | Adhesion G protein-coupled receptor L2 | | |
| rs6460055 | 7q11.23 | 7 | 73,187,517 | 3.65E-06 | *CLDN3* | Claudin 3 |  |  |
| rs1418253 | 1p31.1 | 1 | 82,338,927 | 4.05E-06 | *ADGRL2* | Adhesion G protein-coupled receptor L2 | | |
| **rs17037869** | 2p16.3 | 2 | 49,156,953 | 6.57E-06 | *FSHR* | Follicle stimulating hormone receptor | | |
| rs73290435 | 14q22.3 | 14 | 57,454,032 | 7.73E-06 | *OTX2-AS1* | OTX2 antisense RNA 1 | | |
| **rs13251143** | 8q24.3 | 8 | 140,350,956 | 9.28E-06 | *KCNK9* | Potassium two pore domain channel subfamily K member 9 | | |

**Note**: CHR: chromosome; The content discussed in detail were in bold.
