# Supplementary material for: Heritability and Genome-Wide Association Study of Plasma Cholesterol in Chinese Adult Twins
Source: Front Endocrinol (Lausanne). 2018 Nov 15;9:677. doi: 10.3389/fendo.2018.00677 (PMC6249314; doi:10.3389/fendo.2018.00677)
Supplement: Supplemental Table 7 — The summary of SNPs with P < 1 × 10^−5^ for association with TC in GWAS imputation. [file Table_7.DOCX]

**Supplemental** Table 7 The summary of SNPs with P-value <1×10^-5^ for association with TC in GWAS imputation

| SNP | Chr band | CHR | BP | *P*-value | Closest genes or genes | Official full name |
| --- | --- | --- | --- | --- | --- | --- |
| rs77348447 | 2p16.3 | 2 | 4,823,0828 | 1.705E-07 | *LOC105374591* | Uncharacterized LOC105374591 |
| rs147010738 | 13q21.33 | 13 | 72,767,168 | 1.177E-06 | *DACH1* | Dachshund Family Transcription Factor 1 |
| rs72856182 | 11p15.4 | 11 | 10,466,474 | 1.36E-06 | *AMPD3* | Adenosine monophosphate deaminase 3 |
| rs10840416 | 11p15.4 | 11 | 10,467,504 | 1.808E-06 | *AMPD3* | Adenosine monophosphate deaminase 3 |
| rs10840417 | 11p15.4 | 11 | 10,467,607 | 1.808E-06 | *AMPD3* | Adenosine monophosphate deaminase 3 |
| rs12778233 | 10p13 | 10 | 13,258,320 | 2.223E-06 | *UCMA* | Upper zone of growth plate and  cartilage matrix associated |
| rs7107698 | 11p15.4 | 11 | 10,470,744 | 2.293E-06 | *AMPD3* | Adenosine monophosphate deaminase 3 |
| rs4909929 | 11p15.4 | 11 | 10,471,199 | 2.293E-06 | *AMPD3* | Adenosine monophosphate deaminase 3 |
| rs7937289 | 11p15.4 | 11 | 10,471,931 | 2.293E-06 | *AMPD3* | Adenosine monophosphate deaminase 3 |
| rs12184411 | 11p15.4 | 11 | 10,473,170 | 2.293E-06 | *AMPD3* | Adenosine monophosphate deaminase 3 |
| rs4909928 | 11p15.4 | 11 | 10,470,275 | 2.293E-06 | *AMPD3* | Adenosine monophosphate deaminase 3 |
| rs4910140 | 11p15.4 | 11 | 10,475,196 | 2.293E-06 | *AMPD3* | Adenosine monophosphate deaminase 3 |
| rs112959129 | 7q11.23 | 7 | 73,147,992 | 2.384E-06 | *ABHD11-AS1* | ABHD11 antisense RNA 1 |
| rs10840418 | 11p15.4 | 11 | 10,467,636 | 2.45E-06 | *AMPD3* | Adenosine monophosphate deaminase 3 |
| rs8077755 | 17q21.33 | 17 | 48,634,564 | 2.513E-06 | *CACNA1G-AS1* | CACNA1G antisense RNA 1 |
| rs10709823 | 11p15.4 | 11 | 10,476,526 | 3.869E-06 | *AMPD3* | Adenosine monophosphate deaminase 3 |
| rs7124144 | 11p15.4 | 11 | 10,479,374 | 4.688E-06 | *AMPD3* | Adenosine monophosphate deaminase 3 |
| rs111751846 | 11p15.4 | 11 | 10,479,485 | 4.688E-06 | *AMPD3* | Adenosine monophosphate deaminase 3 |
| rs35815414 | 11p15.4 | 11 | 10,481,139 | 4.688E-06 | *AMPD3* | Adenosine monophosphate deaminase 3 |
| rs7109334 | 11p15.4 | 11 | 10,479,303 | 4.688E-06 | *AMPD3* | Adenosine monophosphate deaminase 3 |
| rs7123980 | 11p15.4 | 11 | 10,479,245 | 4.688E-06 | *AMPD3* | Adenosine monophosphate deaminase 3 |
| rs5005725 | 1p21.1 | 1 | 104,630,807 | 4.79E-06 | *LOC100129138* | THAP domain containing 3 pseudogene |
| rs5005720 | 1p21.1 | 1 | 104,630,904 | 4.79E-06 | *LOC100129138* | THAP domain containing 3 pseudogene |
| rs5005721 | 1p21.1 | 1 | 104,630,890 | 4.79E-06 | *LOC100129138* | THAP domain containing 3 pseudogene |
| rs5005723 | 1p21.1 | 1 | 104,630,842 | 4.79E-06 | *LOC100129138* | THAP domain containing 3 pseudogene |
| rs5005724 | 1p21.1 | 1 | 104,630,836 | 4.79E-06 | *LOC100129138* | THAP domain containing 3 pseudogene |
| rs12490307 | 3q29 | 3 | 195,623,440 | 4.866E-06 | *TNK2* | Tyrosine kinase non receptor 2 |
| rs77828245 | 3q29 | 3 | 195,622,616 | 4.866E-06 | *TNK2* | Tyrosine kinase non receptor 2 |
| rs62283334 | 3q29 | 3 | 195,624,484 | 4.866E-06 | *TNK2* | Tyrosine kinase non receptor 2 |
| rs75683599 | 3q29 | 3 | 195,644,262 | 5.33E-06 | *TNK2-AS1* | TNK2 antisense RNA 1 |
| rs78410324 | 4p14 | 4 | 37,699,846 | 6.057E-06 | *LOC105374404* | Uncharacterized LOC105374404 |
| rs17578959 | 2q36.3 | 2 | 229,877,099 | 6.325E-06 | *PID1* | Phosphotyrosine interaction domain containing 1 |
| rs28845526 | 2q36.3 | 2 | 229,895,007 | 6.971E-06 | *PID1* | Phosphotyrosine interaction domain containing 1 |
| rs59915692 | 7q31.33 | 7 | 125,283,519 | 7.297E-06 | *LOC100506664* | Uncharacterized LOC100506664 |
| rs310892 | 5p14.3 | 5 | 23,195,394 | 7.59E-06 | *LOC105374687* | Uncharacterized LOC105374687 |
| rs13129710 | 4q28.3 | 4 | 138,229,547 | 7.879E-06 | *LOC105377441* | Long intergenic non-protein coding RNA 2511 |
| rs11811543 | 1p21.1 | 1 | 104,625,980 | 8.138E-06 | *LOC100129138* | THAP domain containing 3 pseudogene |
| rs34298462 | 1q43 | 1 | 240,424,651 | 8.162E-06 | *FMN2* | Formin 2 |
| rs1425971 | 4q28.3 | 4 | 138,228,499 | 9.808E-06 | *LOC105377441* | Long intergenic non-protein coding RNA 2511 |

**Note**: kgp, 1000 Genomes Project; CHR, chromosome;
