# Supplementary material for: Heritability and Genome-Wide Association Study of Plasma Cholesterol in Chinese Adult Twins
Source: Front Endocrinol (Lausanne). 2018 Nov 15;9:677. doi: 10.3389/fendo.2018.00677 (PMC6249314; doi:10.3389/fendo.2018.00677)
Supplement: Supplemental Table 8 — The summary of SNPs with P < 1 × 10^−5^ for association with HDL-C in GWAS imputation. [file Table_8.DOCX]

**Supplemental** Table 8 The summary of SNPs with P-value <1×10^-5^ for association with HDL-C in GWAS imputation

| SNP | Chr band | CHR | BP | *P*-value | Closest genes or genes | Official full name | |  |
| --- | --- | --- | --- | --- | --- | --- | --- | --- |
| rs79775842 | 5p15.2 | 5 | 14,318,004 | 3.20549E-07 | *TRIO* | Trio Rho guanine nucleotide exchange factor | | |
| rs79031100 | 5p15.2 | 5 | 14,318,206 | 3.20549E-07 | *TRIO* | Trio Rho guanine nucleotide exchange factor | | |
| rs199929635 | 5q14.1 | 5 | 77,142,788 | 7.10175E-07 | *LOC101929154* | Uncharacterized LOC101929154 | | |
| rs34643831 | 10p13 | 10 | 17,051,623 | 1.40124E-06 | *CUBN* | Cubilin |  |  |
| rs138440365 | 8q22.3 | 8 | 105,243,121 | 1.63389E-06 | *RIMS2* | Regulating synaptic membrane exocytosis 2 | | |
| rs149867584 | 8q22.3 | 8 | 105,245,094 | 1.63389E-06 | *RIMS2* | Regulating synaptic membrane exocytosis 2 | | |
| rs6468909 | 8q22.3 | 8 | 105,227,985 | 1.64576E-06 | *RIMS2* | Regulating synaptic membrane exocytosis 2 | | |
| rs72685070 | 8q22.3 | 8 | 105,250,237 | 1.70647E-06 | *RIMS2* | Regulating synaptic membrane exocytosis 2 | | |
| rs72685059 | 8q22.3 | 8 | 105,238,549 | 1.76423E-06 | *RIMS2* | Regulating synaptic membrane exocytosis 2 | | |
| rs11300039 | 8q22.3 | 8 | 105,235,650 | 1.76423E-06 | *RIMS2* | Regulating synaptic membrane exocytosis 2 | | |
| rs28685021 | 8q22.3 | 8 | 105,241,120 | 1.76423E-06 | *RIMS2* | Regulating synaptic membrane exocytosis 2 | | |
| rs72685060 | 8q22.3 | 8 | 105,238,572 | 1.76423E-06 | *RIMS2* | Regulating synaptic membrane exocytosis 2 | | |
| rs6468910 | 8q22.3 | 8 | 105,229,160 | 1.87329E-06 | *RIMS2* | Regulating synaptic membrane exocytosis 2 | | |
| rs7834250 | 8q22.3 | 8 | 105,231,040 | 1.87329E-06 | *RIMS2* | Regulating synaptic membrane exocytosis 2 | | |
| rs7818442 | 8q22.3 | 8 | 105,233,410 | 1.87329E-06 | *RIMS2* | Regulating synaptic membrane exocytosis 2 | | |
| rs147709455 | 8q22.3 | 8 | 105,250,398 | 1.9495E-06 | *RIMS2* | Regulating synaptic membrane exocytosis 2 | | |
| rs72685066 | 8q22.3 | 8 | 105,248,565 | 1.9495E-06 | *RIMS2* | Regulating synaptic membrane exocytosis 2 | | |
| rs11439544 | 7q31.32 | 7 | 122,811,010 | 2.07614E-06 | *SLC13A1* | Solute carrier family 13 member 1 | | |
| rs12518218 | 5q14.1 | 5 | 77,161,489 | 2.22053E-06 | *LOC101929154* | Uncharacterized LOC101929154 | | |
| rs11218895 | 11q24.1 | 11 | 122,809,690 | 2.40261E-06 | *JHY* | Junctional cadherin complex regulator | | |
| rs4505076 | 11q24.1 | 11 | 122,806,028 | 2.40261E-06 | *JHY* | Junctional cadherin complex regulator | | |
| rs7109006 | 11q24.1 | 11 | 122,809,042 | 2.40261E-06 | *JHY* | Junctional cadherin complex regulator | | |
| rs241178 | 8p21.1 | 8 | 28,626,418 | 2.66922E-06 | *INTS9* | Integrator complex subunit 9 | | |
| rs2711015 | 7p15.3 | 7 | 25,115,316 | 2.70603E-06 | *CYCS* | Cytochrome c, somatic | |  |
| rs143583196 | 17p13.2 | 17 | 5,953,653 | 2.78308E-06 | *WSCD1* | WSC domain containing 1 | |  |
| rs77745521 | 18q11.2 | 18 | 22,956,486 | 3.45857E-06 | *ZNF521* | Zinc finger protein 521 | |  |
| rs17594493 | 4p15.2 | 4 | 24,890,961 | 3.79194E-06 | *CCDC149* | Coiled-coil domain containing 149 | | |
| rs6887636 | 5q14.1 | 5 | 77,137,984 | 4.41458E-06 | *LOC101929154* | Uncharacterized LOC101929154 | | |
| rs59796303 | 4p15.2 | 4 | 24,896,473 | 4.42609E-06 | *CCDC149* | Coiled-coil domain containing 149 | | |
| rs10939012 | 4p15.2 | 4 | 24,897,032 | 4.42609E-06 | *CCDC149* | Coiled-coil domain containing 149 | | |
| rs12414709 | 10p13 | 10 | 17,041,083 | 5.89831E-06 | *CUBN* | Cubilin |  |  |
| rs77208998 | 2q37.3 | 2 | 242,957,107 | 6.01329E-06 | *LINC01237* | Long intergenic non-protein coding RNA 1237 | | |
| rs12511068 | 4p15.2 | 4 | 24,896,658 | 6.03758E-06 | *CCDC149* | Coiled-coil domain containing 149 | | |
| rs7729225 | 5q14.1 | 5 | 77,142,829 | 6.04952E-06 | *LOC101929154* | Uncharacterized LOC101929154 | | |
| rs34942101 | 1q42.11 | 1 | 224,250,053 | 6.32962E-06 | *LOC105373061* | Uncharacterized LOC105373061 | | |
| rs201647698 | 8q22.3 | 8 | 105,243,123 | 6.3644E-06 | *RIMS2* | Regulating synaptic membrane exocytosis 2 | | |
| rs3740373 | 10q23.33 | 10 | 95,256,200 | 6.56533E-06 | *CEP55* | Centrosomal protein 55 | |  |
| rs4697492 | 4p15.2 | 4 | 24,899,304 | 7.43829E-06 | *CCDC149* | Coiled-coil domain containing 149 | | |
| rs61793770 | 4p15.2 | 4 | 24,892,833 | 7.44328E-06 | *CCDC149* | Coiled-coil domain containing 149 | | |
| rs17185061 | 5q14.1 | 5 | 77,139,236 | 7.80426E-06 | *LOC101929154* | Uncharacterized LOC101929154 | | |
| rs201510740 | 1p34.2 | 1 | 40,686,993 | 7.94926E-06 | *RLF* | Rearranged L-myc fusion | |  |
| rs62362869 | 5q14.1 | 5 | 77,156,392 | 9.00072E-06 | *LOC101929154* | Uncharacterized LOC101929154 | | |
| rs115980554 | 6p21.31 | 6 | 34,456,632 | 9.01996E-06 | *PACSIN1* | Protein kinase C and casein kinase substrate in neurons 1 | | |
| rs151103055 | 6p21.31 | 6 | 34,460,168 | 9.01996E-06 | *PACSIN1* | Protein kinase C and casein kinase substrate in neurons 1 | | |
| rs75489661 | 6p21.31 | 6 | 34,458,325 | 9.01996E-06 | *PACSIN1* | Protein kinase C and casein kinase substrate in neurons 1 | | |
| rs62362872 | 5q14.1 | 5 | 77,172,113 | 9.02477E-06 | *LOC101929154* | Uncharacterized LOC101929154 | | |
| rs138464032 | 1q41 | 1 | 221,628,542 | 9.07825E-06 | *LOC105372932* | Uncharacterized LOC105372932 | | |
| rs73405681 | 6p21.31 | 6 | 34,453,626 | 9.24423E-06 | *PACSIN1* | Protein kinase C and casein kinase substrate in neurons 1 | | |
| rs17345993 | 10p13 | 10 | 17,032,885 | 9.56131E-06 | *CUBN* | Cubilin |  |  |
| rs3816870 | 10p13 | 10 | 17,032,834 | 9.56131E-06 | *CUBN* | Cubilin |  |  |
| rs7677838 | 4p15.2 | 4 | 24,898,963 | 9.81383E-06 | *CCDC149* | Coiled-coil domain containing 149 | | |
| rs7897776 | 10q26.2 | 10 | 129,248,408 | 9.8977E-06 | *DOCK1* | Dedicator of cytokinesis 1 | |  |
| rs554384272 | Xq28 | 23 | 150,854,050 | 9.98532E-06 | *PRRG3* | Proline rich and Gla domain 3 | | |
| rs6627443 | Xq28 | 23 | 150,854,327 | 9.98532E-06 | *PRRG3* | Proline rich and Gla domain 3 | | |
| rs6627442 | Xq28 | 23 | 150,852,220 | 9.98532E-06 | *PRRG3* | Proline rich and Gla domain 3 | | |

**Note**: kgp, 1000 Genomes Project; CHR, chromosome;
