# Supplementary material for: Heritability and Genome-Wide Association Study of Plasma Cholesterol in Chinese Adult Twins
Source: Front Endocrinol (Lausanne). 2018 Nov 15;9:677. doi: 10.3389/fendo.2018.00677 (PMC6249314; doi:10.3389/fendo.2018.00677)
Supplement: Supplemental Table 9 — The summary of SNPs with P < 1 × 10^−5^ for association with LDL-C in GWAS imputation. [file Table_9.DOCX]

**Supplemental** Table 9 The summary of SNPs with P-value <1×10^-5^ for association with LDL-C in GWAS imputation

| SNP | Chr band | CHR | BP | *P*-value | Closest genes or genes | Official full name | |
| --- | --- | --- | --- | --- | --- | --- | --- |
| rs56047090 | 1q21.2 | 1 | 150,315,724 | 4.9764E-07 | *PRPF3* | Pre-mRNA processing factor 3 | |
| rs10490120 | 2p16.3 | 2 | 49,143,829 | 1.1097E-06 | *FSHR* | Follicle stimulating hormone receptor | |
| rs112959129 | 7q11.23 | 7 | 73,147,992 | 1.2791E-06 | *ABHD11-AS1* | ABHD11 antisense RNA 1 | |
| rs4953640 | 2p16.3 | 2 | 49,126,802 | 1.321E-06 | *FSHR* | Follicle stimulating hormone receptor | |
| rs17038437 | 2p16.3 | 2 | 49,142,398 | 1.4661E-06 | *FSHR* | Follicle stimulating hormone receptor | |
| rs4953642 | 2p16.3 | 2 | 49,139,672 | 1.4866E-06 | *FSHR* | Follicle stimulating hormone receptor | |
| rs4870470 | 6q25.3 | 6 | 156,914,985 | 1.5395E-06 | *ARID1B* | AT-rich interaction domain 1B | |
| rs74263479 | 2p16.3 | 2 | 49,132,194 | 1.6701E-06 | *FSHR* | Follicle stimulating hormone receptor | |
| rs10709251 | 7q11.23 | 7 | 73,189,060 | 1.7532E-06 | *CLDN3* | Claudin 3 |  |
| rs45524931 | 2p16.3 | 2 | 49,131,696 | 1.8494E-06 | *FSHR* | Follicle stimulating hormone receptor | |
| rs367881 | 1p31.1 | 1 | 82,348,717 | 2.0389E-06 | *ADGRL2* | Adhesion G protein-coupled receptor L2 | |
| rs74849818 | 2p16.3 | 2 | 49,125,706 | 2.144E-06 | *FSHR* | Follicle stimulating hormone receptor | |
| rs79436813 | 2p16.3 | 2 | 49,125,876 | 2.2601E-06 | *FSHR* | Follicle stimulating hormone receptor | |
| rs4953644 | 2p16.3 | 2 | 49,155,400 | 2.5133E-06 | *FSHR* | Follicle stimulating hormone receptor | |
| rs141507877 | 3q29 | 3 | 195,621,049 | 3.0209E-06 | *TNK2* | Tyrosine kinase non receptor 2 | |
| rs6460055 | 7q11.23 | 7 | 73,187,517 | 3.6515E-06 | *CLDN3* | Claudin 3 |  |
| rs6958663 | 7q11.23 | 7 | 73,189,302 | 3.6515E-06 | *CLDN3* | Claudin 3 |  |
| rs11979680 | 7q11.23 | 7 | 73,188,574 | 3.6515E-06 | *CLDN3* | Claudin 3 |  |
| rs113087245 | 7q11.23 | 7 | 73,189,775 | 3.6515E-06 | *CLDN3* | Claudin 3 |  |
| rs10236342 | 7q11.23 | 7 | 73,189,279 | 3.6515E-06 | *CLDN3* | Claudin 3 |  |
| rs66516150 | 1p31.1 | 1 | 82,345,197 | 4.0525E-06 | *ADGRL2* | Adhesion G protein-coupled receptor L2 | |
| rs1418253 | 1p31.1 | 1 | 82,338,927 | 4.0525E-06 | *ADGRL2* | Adhesion G protein-coupled receptor L2 | |
| rs140385795 | 1p31.1 | 1 | 82,337,278 | 4.0525E-06 | *ADGRL2* | Adhesion G protein-coupled receptor L2 | |
| rs1612916 | 1p31.1 | 1 | 82,340,360 | 4.0525E-06 | *ADGRL2* | Adhesion G protein-coupled receptor L2 | |
| rs11356912 | 1p31.1 | 1 | 82,343,392 | 4.0525E-06 | *ADGRL2* | Adhesion G protein-coupled receptor L2 | |
| rs284230 | 1p31.1 | 1 | 82,344,854 | 4.0525E-06 | *ADGRL2* | Adhesion G protein-coupled receptor L2 | |
| rs284231 | 1p31.1 | 1 | 82,344,514 | 4.0525E-06 | *ADGRL2* | Adhesion G protein-coupled receptor L2 | |
| rs12144126 | 1p31.1 | 1 | 82,336,922 | 4.0525E-06 | *ADGRL2* | Adhesion G protein-coupled receptor L2 | |
| rs76599784 | 2p16.3 | 2 | 49,147,108 | 4.4435E-06 | *FSHR* | Follicle stimulating hormone receptor | |
| rs12925859 | 16q12.1 | 16 | 52,227,939 | 4.4549E-06 | *LOC105371261* | Uncharacterized LOC105371261 | |
| rs12139220 | 1p31.1 | 1 | 82,327,159 | 4.5137E-06 | *ADGRL2* | Adhesion G protein-coupled receptor L2 | |
| rs77348447 | 2p16.3 | 2 | 48,230,828 | 4.6675E-06 | *LOC105374591* | Uncharacterized LOC105374591 | |
| rs406456 | 19q13.32 | 19 | 45,382,717 | 4.9548E-06 | *NECTIN2* | Nectin cell adhesion molecule 2 | |
| rs7782126 | 7q11.23 | 7 | 73,154,074 | 5.0723E-06 | *ABHD11* | Abhydrolase domain containing 11 | |
| rs4588831 | 8q24.3 | 8 | 140,350,233 | 5.1522E-06 | *KCNK9* | Potassium two pore domain channel subfamily K member 9 | |
| rs151219533 | 2p16.3 | 2 | 49,152,371 | 5.7401E-06 | *FSHR* | Follicle stimulating hormone receptor | |
| rs77828245 | 3q29 | 3 | 195,622,616 | 5.9692E-06 | *TNK2* | Tyrosine kinase non receptor 2 | |
| rs12490307 | 3q29 | 3 | 195,623,440 | 5.9692E-06 | *TNK2* | Tyrosine kinase non receptor 2 | |
| rs62283334 | 3q29 | 3 | 195,624,484 | 5.9692E-06 | *TNK2* | Tyrosine kinase non receptor 2 | |
| rs10709722 | 7q11.23 | 7 | 73,183,271 | 6.5597E-06 | *CLDN3* | Claudin 3 |  |
| rs17037869 | 2p16.3 | 2 | 49,156,953 | 6.5651E-06 | *FSHR* | Follicle stimulating hormone receptor | |
| rs34994196 | 19q13.32 | 19 | 45,384,332 | 6.7269E-06 | *NECTIN2* | Nectin cell adhesion molecule 2 | |
| rs57618243 | 14q22.3 | 14 | 57,450,864 | 7.0931E-06 | *RPL3P3* | Ribosomal protein L3 pseudogene 3 | |
| rs4717106 | 7q11.23 | 7 | 73,172,292 | 7.2217E-06 | *CLDN3* | Claudin 3 |  |
| rs10949827 | 7q11.23 | 7 | 73,172,186 | 7.2217E-06 | *CLDN3* | Claudin 3 |  |
| rs73290435 | 14q22.3 | 14 | 57,454,032 | 7.7311E-06 | *RPL3P3* | Ribosomal protein L3 pseudogene 3 | |
| rs11981405 | 7q11.23 | 7 | 73,162,437 | 8.1235E-06 | *ABHD11* | Abhydrolase domain containing 11 | |
| rs1880953 | 7q11.23 | 7 | 73,157,882 | 8.1235E-06 | *ABHD11* | Abhydrolase domain containing 11 | |
| rs12539962 | 7q11.23 | 7 | 73,167,259 | 8.6812E-06 | *ABHD11* | Abhydrolase domain containing 11 | |
| rs12146061 | 1p31.1 | 1 | 82,326,742 | 8.8946E-06 | *ADGRL2* | Adhesion G protein-coupled receptor L2 | |
| rs377752731 | 9q32 | 9 | 116,596,245 | 9.2218E-06 | *ZNF618* | Zinc finger protein 618 | |
| rs13251143 | 8q24.3 | 8 | 140,350,956 | 9.2842E-06 | *KCNK9* | Potassium two pore domain channel subfamily K member 9 | |
| rs7154844 | 14q22.3 | 14 | 57,456,220 | 9.4284E-06 | *RPL3P3* | Ribosomal protein L3 pseudogene 3 | |
| rs10132510 | 14q22.3 | 14 | 57,458,578 | 9.4284E-06 | *RPL3P3* | Ribosomal protein L3 pseudogene 3 | |
| rs2134921 | 14q22.3 | 14 | 57,458,566 | 9.4284E-06 | *RPL3P3* | Ribosomal protein L3 pseudogene 3 | |
| rs61348921 | 14q22.3 | 14 | 57,457,805 | 9.4284E-06 | *RPL3P3* | Ribosomal protein L3 pseudogene 3 | |
| rs1985129 | 19q13.32 | 19 | 45,384,338 | 9.7356E-06 | *NECTIN2* | Nectin cell adhesion molecule 2 | |
| rs56716122 | 6q21 | 6 | 113,780,613 | 9.7384E-06 | *LINC02518* | Long intergenic non-protein coding RNA 2518 | |
| rs67231016 | 1p31.1 | 1 | 82,335,390 | 9.8021E-06 | *ADGRL2* | Adhesion G protein-coupled receptor L2 | |

**Note**: kgp, 1000 Genomes Project; CHR, chromosome;
