# Supplementary material for: Heritability and Genome-Wide Association Study of Plasma Cholesterol in Chinese Adult Twins
Source: Front Endocrinol (Lausanne). 2018 Nov 15;9:677. doi: 10.3389/fendo.2018.00677 (PMC6249314; doi:10.3389/fendo.2018.00677)
Supplement: Supplemental Table 10 — The comparison between our imputation results of genome-wide association study and other East Asian studies. [file Table_10.DOCX]

**Supplemental Table 10** The comparison between our imputation results of genome-wide association study and other East Asian studies

| Trait | SNP | Gene | Chr | BP | Our study* | |  | Other east Asian studies# | | | |  |
| --- | --- | --- | --- | --- | --- | --- | --- | --- | --- | --- | --- | --- |
|  |  |  |  |  | N | Beta | *P*-value | N | Beta | *P*-value | |  |
| TC | rs17357348 | *RGS5* | 1 | 163103939 | 277 | 0.6341593 | 6.78E-04 | 15,153 | -0.0643 | 9.42E-03 | |  |
| TC | rs12408821 | *RGS5* | 1 | 163109029 | 278 | 0.6223461 | 7.11E-04 | 15,155 | -0.0645 | 9.10E-03 |  |  |
| TC | rs10917682 | *RGS5* | 1 | 163112204 | 276 | 0.6022006 | 1.31E-03 | 15,197 | -0.0642 | 9.37E-03 | | |
| TC | rs3806405 | *HECTD3/UROD* | 1 | 45476489 | 265 | -0.3523721 | 7.22E-04 | 10,133 | 0.0433 | 7.57E-03 | | |
| TC | rs7340001 | *LINC01141* | 1 | 20705948 | 267 | 0.3252348 | 9.42E-04 | 20,899 | -0.0273 | 9.70E-03 | | |
| TC | rs12035395 | *PTGER3* | 1 | 71368753 | 275 | 0.4046184 | 1.57E-03 | 17,072 | -0.0415 | 4.60E-03 | | |
| TC | rs17038052 | *LOC105376744* | 1 | 12587934 | 277 | -0.4475915 | 1.89E-03 | 13,083 | -0.0784 | 6.19E-03 | | |
| TC | rs1179723 | *LOC101928278/*  *LOC105373876* | 2 | 217733190 | 278 | -0.3142924 | 1.27E-03 | 24,041 | -0.025 | 7.80E-03 | | |
| TC | rs1542354 | *LOC107985988* | 2 | 221814085 | 277 | 0.3048427 | 1.58E-03 | 21,734 | -0.0353 | 9.73E-04 | | |
| TC | rs936160 | *OSBPL10* | 3 | 31812585 | 264 | -0.3937993 | 1.33E-03 | 32,017 | 0.0309 | 4.17E-03 | | |
| TC | rs2068100 | *OSBPL10* | 3 | 31837204 | 273 | -0.4066185 | 1.50E-03 | 31,817 | -0.0282 | 9.42E-03 | | |
| TC | rs9846001 | *FOXP1* | 3 | 71660196 | 278 | -0.4704912 | 1.55E-03 | 5,764 | -0.1074 | 3.15E-03 | | |
| TC | rs2686099 | *LOC105374308* | 3 | 197193567 | 276 | -0.461137 | 1.90E-03 | 28,927 | -0.0445 | 9.59E-03 | | |
| TC | rs12173971 | *ADGRB3* | 6 | 70092257 | 277 | -0.4513927 | 1.48E-03 | 31,732 | -0.0446 | 7.06E-04 | | |
| TC | rs9351751 | *ADGRB3* | 6 | 70088968 | 276 | -0.4473911 | 1.63E-03 | 31,740 | 0.0448 | 6.59E-04 | | |
| TC | rs9346273 | *ADGRB3* | 6 | 70104023 | 277 | -0.4445636 | 1.64E-03 | 32,005 | -0.0405 | 2.01E-03 | | |
| TC | rs12525747 | *ADGRB3* | 6 | 70102584 | 277 | -0.4445636 | 1.64E-03 | 31,829 | 0.0389 | 3.56E-03 | | |
| TC | rs2797369 | *LOC107984041* | 6 | 101674290 | 268 | -0.3290027 | 1.67E-03 | 11,535 | 0.0437 | 7.23E-03 | | |
| TC | rs4416845 | *LOC107986941* | 8 | 49120572 | 271 | 0.2996996 | 1.65E-03 | 31,955 | 0.0234 | 4.33E-03 | | |
| TC | rs4242486 | *LOC100287157* | 8 | 49116501 | 273 | 0.2962427 | 1.86E-03 | 28,143 | 0.0271 | 2.02E-03 | | |
| TC | rs7838270 | *LOC100287157* | 8 | 49112845 | 273 | 0.2962427 | 1.86E-03 | 31,970 | -0.0233 | 4.57E-03 | | |
| TC | rs4838170 | *PSMB7* | 9 | 127159770 | 275 | -0.3033051 | 1.47E-03 | 31,792 | 0.0245 | 5.08E-03 | | |
| TC | rs7047609 | *PSMB7* | 9 | 127122758 | 277 | -0.3002859 | 1.70E-03 | 31,709 | 0.0239 | 4.88E-03 | | |
| TC | rs3758208 | *PSMB7* | 9 | 127126278 | 277 | -0.3002859 | 1.70E-03 | 30,725 | -0.0227 | 8.59E-03 | | |
| TC | rs681695 | *SRSF8* | 11 | 94802776 | 278 | -0.3184424 | 7.19E-04 | 30,357 | -0.0277 | 5.45E-03 | | |
| TC | rs653573 | *SRSF8* | 11 | 94804852 | 277 | -0.3057284 | 1.18E-03 | 30,692 | -0.0269 | 6.79E-03 | | |
| TC | rs12574397 | *RNU6-544P* | 11 | 80267205 | 276 | -0.3739294 | 1.50E-03 | 31,960 | 0.0269 | 6.91E-03 | | |
| TC | rs17143789 | *LOC105369410* | 11 | 82248807 | 272 | 0.7892496 | 1.68E-03 | 3,827 | -0.105 | 8.95E-03 | | |
| TC | rs6505064 | *NOG* | 17 | 54696955 | 278 | -0.6369583 | 1.17E-03 | 24,003 | -0.0395 | 8.10E-03 | | |
| TC | rs283813 | *NECTIN2* | 19 | 45389174 | 277 | -0.4972751 | 9.83E-04 | 15,874 | -0.2953 | 4.01E-41 | | |
| TC | rs427736 | *MN1* | 22 | 28173898 | 278 | 0.3791952 | 9.25E-04 | 11,060 | -0.0457 | 3.40E-03 | | |
| TC | rs132857 | *NUP50-AS1/NUP50* | 22 | 45561392 | 275 | 0.2888002 | 1.67E-03 | 21,437 | 0.0367 | 1.09E-03 | | |
| HDL | rs1416624 | *KAZN* | 1 | 14629604 | 278 | -0.2996963 | 9.43E-04 | 11,051 | -0.0461 | 9.57E-04 | | |
| HDL | rs11589133 | *KAZN* | 1 | 14630326 | 276 | -0.2964365 | 1.08E-03 | 11,054 | -0.0451 | 1.34E-03 | | |
| HDL | rs1416625 | *KAZN* | 1 | 14629449 | 278 | -0.3012571 | 1.63E-03 | 11,051 | 0.0507 | 6.47E-04 | | |
| HDL | rs6667154 | *LOC105378657* | 1 | 38849805 | 278 | 0.406012 | 1.50E-03 | 34,078 | -0.0313 | 8.11E-03 | | |
| HDL | rs17538148 | *LOC105378657* | 1 | 38858640 | 278 | 0.406012 | 1.50E-03 | 34,061 | -0.0317 | 7.47E-03 | | |
| HDL | rs12740005 | *LOC105378657* | 1 | 38858540 | 278 | 0.406012 | 1.50E-03 | 34,060 | -0.0313 | 7.97E-03 | | |
| HDL | rs6677719 | *RPL27P2* | 1 | 159723120 | 278 | -0.38979 | 1.99E-03 | 30,298 | -0.0329 | 4.86E-03 | | |
| HDL | rs4355090 | *ICOS* | 2 | 204816382 | 278 | 0.2871189 | 2.81E-04 | 2,179 | 0.0852 | 8.50E-03 | | |
| HDL | rs1607420 | *LOC105373529* | 2 | 106097519 | 278 | 0.2839034 | 7.34E-04 | 21,740 | 0.0277 | 7.71E-03 | | |
| HDL | rs12988002 | *LOC105373529* | 2 | 106097275 | 275 | 0.2823052 | 7.93E-04 | 21,740 | 0.0272 | 9.14E-03 | | |
| HDL | rs2048982 | *LOC105373529* | 2 | 106090331 | 278 | 0.2802042 | 9.01E-04 | 25,577 | -0.0264 | 5.61E-03 | | |
| HDL | rs4663195 | *LOC105373941* | 2 | 236250584 | 277 | 0.258918 | 1.47E-03 | 17,618 | -0.0312 | 2.44E-03 | | |
| HDL | rs6728599 | *LOC105373941* | 2 | 236248940 | 277 | 0.258918 | 1.47E-03 | 17,618 | 0.031 | 2.52E-03 | | |
| HDL | rs9287590 | *LOC105373941* | 2 | 236252043 | 278 | 0.2580542 | 1.57E-03 | 17,622 | 0.0306 | 3.05E-03 | | |
| HDL | rs10175710 | *LOC105373941* | 2 | 236251043 | 278 | 0.2580542 | 1.57E-03 | 15,565 | -0.033 | 2.88E-03 | | |
| HDL | rs6431368 | *LOC105373941* | 2 | 236252473 | 275 | 0.2553297 | 1.79E-03 | 17,622 | -0.0307 | 2.87E-03 | | |
| HDL | rs10007440 | *LOC105377468* | 4 | 146987110 | 278 | 0.3360131 | 5.30E-04 | 34,359 | -0.0257 | 7.60E-03 | | |
| HDL | rs1426887 | *LOC105377468* | 4 | 146986231 | 278 | 0.3360131 | 5.30E-04 | 34,352 | -0.0257 | 7.62E-03 | | |
| HDL | rs6812643 | *JAKMIP1* | 4 | 6152171 | 277 | -0.3447389 | 6.28E-04 | 34,369 | 0.0298 | 3.96E-03 | | |
| HDL | rs9312664 | *NMU* | 4 | 56499826 | 277 | 0.319672 | 1.42E-03 | 20,460 | 0.0295 | 5.00E-03 | | |
| HDL | rs17171742 | *LOC100506725* | 7 | 35755836 | 278 | 0.2754477 | 1.07E-03 | 34,349 | 0.0262 | 1.78E-03 | | |
| HDL | rs7778558 | *LOC100506725* | 7 | 35748813 | 278 | 0.2754477 | 1.07E-03 | 33,075 | -0.0231 | 6.37E-03 | | |
| HDL | rs17171719 | *LOC101930085* | 7 | 35744471 | 278 | 0.2754477 | 1.07E-03 | 34,369 | -0.0258 | 1.96E-03 | | |
| HDL | rs4872526 | *BIN3* | 8 | 22495966 | 275 | -0.3479072 | 6.25E-05 | 34,084 | 0.0212 | 7.38E-03 | | |
| HDL | rs7459660 | *BIN3* | 8 | 22505093 | 277 | -0.3010171 | 2.76E-04 | 34,210 | 0.0226 | 4.57E-03 | | |
| HDL | rs9802033 | *BIN3* | 8 | 22503504 | 275 | -0.2912141 | 4.28E-04 | 34,180 | -0.0225 | 4.79E-03 | | |
| HDL | rs1869 | *BIN3* | 8 | 22477585 | 278 | -0.2814895 | 7.42E-04 | 32,314 | -0.0271 | 1.31E-03 | | |
| HDL | rs4872529 | *BIN3/*  *LOC105369180* | 8 | 22513346 | 269 | -0.2910316 | 5.00E-04 | 34,336 | -0.0224 | 5.22E-03 | | |
| HDL | rs13249240 | *LINC02153* | 8 | 20941026 | 259 | 0.2981465 | 1.03E-04 | 25,573 | 0.025 | 6.26E-03 | | |
| HDL | rs4736719 | *LINC02153* | 8 | 20942007 | 262 | 0.2928371 | 1.16E-04 | 25,574 | -0.0252 | 5.89E-03 | | |
| HDL | rs1589457 | *LINC02153* | 8 | 20941808 | 262 | 0.2928371 | 1.16E-04 | 25,574 | 0.0251 | 6.09E-03 | | |
| HDL | rs2013243 | *LINC02153* | 8 | 20924956 | 255 | 0.2869335 | 3.05E-04 | 25,571 | 0.0258 | 5.30E-03 | | |
| HDL | rs11204158 | *LINC02153* | 8 | 20942928 | 265 | 0.2761543 | 3.06E-04 | 34,280 | -0.0224 | 5.06E-03 | | |
| HDL | rs6985275 | *LINC02153* | 8 | 20944251 | 264 | 0.2754391 | 3.18E-04 | 34,352 | -0.023 | 4.06E-03 | | |
| HDL | rs1949640 | *LINC02153* | 8 | 20939367 | 277 | 0.2703844 | 3.25E-04 | 25,571 | -0.0255 | 5.48E-03 | | |
| HDL | rs13250583 | *LINC02153* | 8 | 20949917 | 274 | 0.2725166 | 3.38E-04 | 34,245 | -0.0241 | 2.64E-03 | | |
| HDL | rs11784441 | *LINC02153* | 8 | 20949526 | 274 | 0.2725166 | 3.38E-04 | 34,070 | 0.0238 | 3.08E-03 | | |
| HDL | rs7839300 | *LINC02153* | 8 | 20950485 | 278 | 0.2718669 | 3.49E-04 | 34,369 | 0.0224 | 4.97E-03 | | |
| HDL | rs10111769 | *LINC02153* | 8 | 20958756 | 271 | 0.270071 | 4.87E-04 | 34,371 | 0.0206 | 9.30E-03 | | |
| HDL | rs898648 | *LINC02153* | 8 | 20960490 | 278 | 0.2607787 | 7.39E-04 | 25,575 | 0.0254 | 4.36E-03 | | |
| HDL | rs10503698 | *LINC02153* | 8 | 20979708 | 262 | 0.2428697 | 1.88E-03 | 23,558 | 0.0312 | 1.17E-03 | | |
| HDL | rs10827617 | *LOC107984222* | 10 | 36471685 | 261 | 0.2684464 | 1.57E-03 | 34,346 | -0.0294 | 6.28E-04 | | |
| HDL | rs2075291 | *APOA5/ZPR1* | 11 | 116661392 | 249 | -0.5088327 | 1.75E-03 | 21,741 | -0.3193 | 1.16E-53 | | |
| HDL | rs11174748 | *LDHAL6CP* | 12 | 63405161 | 278 | 0.3237974 | 3.07E-04 | 34,266 | -0.0216 | 8.57E-03 | | |
| HDL | rs11174747 | *LDHAL6CP* | 12 | 63404105 | 278 | 0.2980089 | 9.61E-04 | 34,291 | 0.0214 | 9.38E-03 | | |
| HDL | rs869518 | *LDHAL6CP* | 12 | 63412321 | 278 | 0.2927056 | 1.27E-03 | 34,272 | 0.0218 | 8.08E-03 | | |
| HDL | rs9511658 | *LOC105370120* | 13 | 25713645 | 263 | 0.3643056 | 9.70E-04 | 24,778 | 0.0364 | 1.76E-03 | | |
| HDL | rs17105012 | *RN7SKP17* | 14 | 77375691 | 265 | -0.2591422 | 1.89E-03 | 19,967 | 0.0317 | 5.41E-03 | | |
| HDL | rs41424644 | *RGMA* | 15 | 93598267 | 275 | -0.314752 | 1.14E-03 | 19,004 | -0.039 | 4.04E-03 | | |
| HDL | rs10407344 | *ZNF536* | 19 | 30918305 | 268 | -0.3917253 | 5.65E-04 | 25,576 | -0.0309 | 5.93E-03 | | |
| LDL | rs6660365 | *VPS13D* | 1 | 12480504 | 277 | -0.4815824 | 1.29E-03 | 10,133 | -0.0531 | 8.52E-03 | | |
| LDL | rs34820623 | *VPS13D* | 1 | 12468119 | 278 | -0.4780855 | 1.37E-03 | 10,133 | 0.0542 | 7.53E-03 | | |
| LDL | rs6541020 | *VPS13D* | 1 | 12465147 | 278 | -0.4780855 | 1.37E-03 | 10,133 | 0.0542 | 8.11E-03 | | |
| LDL | rs6696289 | *APOE* | 1 | 244343876 | 278 | 0.3472372 | 1.37E-03 | 30,215 | -0.0261 | 3.00E-03 | | |
| LDL | rs7580273 | *NAGK* | 2 | 71276698 | 271 | 0.4683241 | 4.46E-04 | 23,190 | -0.0415 | 9.36E-04 | | |
| LDL | rs10803928 | *LOC107985967/ ZNF385B* | 2 | 180570920 | 278 | -0.3328985 | 1.37E-03 | 21,711 | 0.0343 | 3.21E-03 | | |
| LDL | rs9859406 | *IGF2BP2* | 3 | 185534482 | 277 | 0.3780965 | 1.33E-03 | 30,448 | 0.0286 | 1.88E-03 | | |
| LDL | rs11705729 | *IGF2BP2* | 3 | 185507299 | 276 | 0.3739247 | 1.53E-03 | 31,707 | -0.0254 | 4.32E-03 | | |
| LDL | rs1470579 | *IGF2BP2* | 3 | 185529080 | 278 | 0.3728213 | 1.55E-03 | 31,709 | -0.0244 | 5.93E-03 | | |
| LDL | rs11711477 | *IGF2BP2* | 3 | 185526690 | 278 | 0.3728213 | 1.55E-03 | 31,709 | -0.0244 | 5.93E-03 | | |
| LDL | rs1470580 | *IGF2BP2* | 3 | 185529174 | 278 | 0.3728213 | 1.55E-03 | 29,982 | 0.0257 | 4.95E-03 | | |
| LDL | rs6769511 | *IGF2BP2* | 3 | 185530290 | 278 | 0.3728213 | 1.55E-03 | 31,729 | -0.0243 | 6.11E-03 | | |
| LDL | rs7633675 | *IGF2BP2* | 3 | 185510613 | 278 | 0.3728213 | 1.55E-03 | 31,708 | -0.0259 | 3.65E-03 | | |
| LDL | rs1643028 | *LOC107986061* | 3 | 8852869 | 275 | 0.3256315 | 1.86E-03 | 31,725 | 0.021 | 9.35E-03 | | |
| LDL | rs1281093 | *SH3TC1* | 4 | 8234904 | 270 | 0.3966123 | 2.73E-04 | 11,527 | -0.0474 | 6.79E-03 | | |
| LDL | rs4838170 | *PSMB7* | 9 | 127159770 | 275 | -0.3200651 | 1.56E-03 | 31,511 | 0.0249 | 4.32E-03 | | |
| LDL | rs3758208 | *PSMB7* | 9 | 127126278 | 277 | -0.3204988 | 1.61E-03 | 30,447 | -0.0251 | 3.69E-03 | | |
| LDL | rs7047609 | *PSMB7* | 9 | 127122758 | 277 | -0.3204988 | 1.61E-03 | 31,439 | 0.0258 | 2.54E-03 | | |
| LDL | rs10116216 | *RASEF* | 9 | 85663674 | 278 | -0.3039475 | 1.79E-03 | 10,132 | 0.0431 | 5.84E-03 | | |
| LDL | rs1924603 | *LINC00428* | 13 | 43418983 | 278 | -0.3576553 | 5.98E-04 | 11,527 | -0.0394 | 5.38E-03 | | |
| LDL | rs1384938 | *LRFNS* | 14 | 42832932 | 274 | 0.687471 | 5.20E-04 | 10,809 | -0.0779 | 7.36E-03 | | |
| LDL | rs9915707 | *CA10* | 17 | 49849336 | 242 | 0.3959068 | 1.57E-04 | 23,190 | 0.0275 | 4.66E-03 | | |
| LDL | rs8072848 | *CA10* | 17 | 49840542 | 278 | 0.3632446 | 2.97E-04 | 23,187 | 0.0262 | 6.39E-03 | | |
| LDL | rs1534203 | *CA10* | 17 | 49846031 | 278 | 0.3379209 | 6.14E-04 | 23,190 | 0.0282 | 3.42E-03 | | |
| LDL | rs8182237 | *CA10* | 17 | 49842542 | 276 | 0.3270763 | 9.91E-04 | 23,189 | 0.0284 | 3.16E-03 | | |
| LDL | rs7254892 | *NECTIN2* | 19 | 45389596 | 278 | -0.7752744 | 3.70E-05 | 14,885 | -0.6405 | 3.20E-127 | | |
| LDL | rs3852861 | *NECTIN2* | 19 | 45383061 | 272 | -0.4710691 | 2.41E-04 | 17,152 | 0.0788 | 5.64E-08 | | |
| LDL | rs283813 | *NECTIN2* | 19 | 45389174 | 277 | -0.6601079 | 3.41E-05 | 15,874 | -0.4851 | 2.83E-106 | | |
| LDL | rs387976 | *NECTIN2* | 19 | 45379060 | 278 | -0.3423827 | 1.36E-03 | 21,713 | -0.1293 | 3.23E-26 | | |
| LDL | rs7412 | *APOE* | 19 | 45412079 | 278 | -0.7147316 | 3.52E-05 | 10,133 | -0.5934 | 8.20E-107 | | |
| LDL | rs405509 | *APOE* | 19 | 45408836 | 274 | -0.359747 | 7.08E-04 | 17,155 | 0.1624 | 4.08E-42 | | |
| LDL | rs439401 | *APOE* | 19 | 45414451 | 273 | -0.3453967 | 7.85E-04 | 17,154 | 0.0725 | 1.09E-10 | | |
| LDL | rs584007 | *APOC1* | 19 | 45416478 | 277 | -0.3426619 | 8.71E-04 | 10,131 | 0.0855 | 1.54E-08 | | |
| LDL | rs445925 | *APOC1* | 19 | 45415640 | 278 | -0.7159951 | 1.88E-05 | 15,874 | -0.5219 | 1.79E-129 | | |
| LDL | rs157582 | *TOMM40* | 19 | 45396219 | 278 | -0.4163587 | 1.34E-03 | 17,155 | -0.1189 | 5.26E-16 | | |
| LDL | rs157590 | *TOMM40* | 19 | 45398716 | 261 | -0.3683223 | 5.01E-04 | 10,133 | 0.1729 | 3.31E-27 | | |
| LDL | rs16994582 | *PLCB1* | 20 | 8250737 | 274 | -0.4221805 | 4.07E-04 | 20,411 | 0.0355 | 9.65E-03 | | |
| LDL | rs6072089 | *LOC102724968* | 20 | 39288335 | 241 | -0.6432427 | 1.75E-03 | 20,485 | 0.0526 | 5.32E-03 | | |
| LDL | rs400406 | *TSPEAR* | 21 | 46016965 | 277 | 0.3771809 | 5.59E-04 | 28,339 | -0.0272 | 6.77E-03 | | |

Note: * We performed the genome-wide association study for plasma cholesterol in a sample of 139 pairs Chinese adult twins.

# A GWAS meta-analysis for HDL-C, LDL-C and TC was conducted in 34,421 East Asians.

Summary statistics for the East Asian lipids meta-analyses are available at: <https://blog.nus.edu.sg/agen/summary-statistics/>
