# Supplementary material for: Heritability and Genome-Wide Association Study of Plasma Cholesterol in Chinese Adult Twins
Source: Front Endocrinol (Lausanne). 2018 Nov 15;9:677. doi: 10.3389/fendo.2018.00677 (PMC6249314; doi:10.3389/fendo.2018.00677)
Supplement: Supplemental Table 11 — The top 20 genes from VEGAS2 gene-based analysis showing the strongest association with TC level in typed GWAS data. [file Table_11.DOCX]

**Supplemental Table 11** The top 20 genes from VEGAS2 gene-based analysis showing the strongest association with TC level in typed GWAS data

| Chr | Gene | Numbers of SNP | Start position | Stop position | Gene-based test statistic | Gene  *P* value | Top-SNP | Top-SNP  *P* value |
| --- | --- | --- | --- | --- | --- | --- | --- | --- |
| 6 | *TMEM14A* | 12 | 52,535,883 | 52,551,385 | 139.39 | 4.80E-05 | rs2670153 | 2.05E-05 |
| 12 | *MLEC* | 22 | 121,124,948 | 121,139,667 | 152.72 | 8.90E-05 | rs73225026 | 6.61E-04 |
| 20 | *ZNF337* | 10 | 25,653,830 | 25,677,540 | 73.93 | 1.20E-04 | rs926487 | 7.92E-04 |
| 2 | *REG1B* | 3 | 79,312,148 | 79,315,150 | 30.84 | 1.70E-04 | rs3739144 | 7.06E-04 |
| 5 | *GPR151* | 3 | 145,894,416 | 145,895,676 | 21.06 | 2.00E-04 | rs7713676 | 1.13E-03 |
| 9 | *KCNV2* | 13 | 2,717,525 | 2,730,037 | 95.39 | 2.00E-04 | rs12352254 | 1.67E-04 |
| 8 | *TRMT12* | 3 | 125,463,047 | 125,465,266 | 20.64 | 2.10E-04 | rs3812475 | 1.32E-03 |
| 12 | *LOC101927583* | 3 | 58,012,187 | 58,015,686 | 27.34 | 2.30E-04 | rs923828 | 5.12E-05 |
| 1 | *HECTD3* | 6 | 45,468,219 | 45,477,027 | 45.58 | 2.50E-04 | rs7541207 | 2.76E-04 |
| 12 | *SLC26A10* | 5 | 58,013,692 | 58,019,934 | 35.54 | 2.60E-04 | rs923828 | 5.12E-05 |
| 10 | *HNRNPA3P1* | 3 | 44,282,859 | 44,285,865 | 21.46 | 2.70E-04 | rs7093194 | 3.88E-05 |
| 1 | *CD1E* | 3 | 158,323,485 | 158,327,343 | 21.09 | 3.10E-04 | rs1065457 | 1.01E-03 |
| 1 | ***FAF1*** | 78 | 50,906,934 | 51,425,936 | 556.47 | 3.30E-04 | rs12084054 | 1.03E-04 |
| 19 | *PPP2R1A* | 27 | 52,693,054 | 52,729,678 | 168.84 | 4.00E-04 | rs8103874 | 5.47E-05 |
| 9 | *FXN* | 39 | 71,650,478 | 71,715,094 | 278.02 | 4.10E-04 | rs2498431 | 7.20E-05 |
| 11 | *PRSS23* | 5 | 86,511,490 | 86,522,273 | 43.54 | 4.10E-04 | rs2155080 | 1.65E-04 |
| 1 | *UROD* | 4 | 45,477,804 | 45,481,341 | 26.82 | 4.80E-04 | rs11211066 | 2.88E-04 |
| 7 | *HYAL4* | 7 | 123,485,222 | 123,517,531 | 46.42 | 5.20E-04 | rs10276917 | 2.28E-04 |
| 4 | ***KLKB1*** | 19 | 187,148,671 | 187,179,625 | 91.09 | 5.30E-04 | rs1912826 | 1.48E-03 |
| 11 | *SRSF8* | 3 | 94,800,040 | 94,804,387 | 26.50 | 5.60E-04 | rs12627 | 7.19E-04 |

**Note**: Chr, chromosome; The content discussed in detail were in bold.
