# Supplementary material for: Heritability and Genome-Wide Association Study of Plasma Cholesterol in Chinese Adult Twins
Source: Front Endocrinol (Lausanne). 2018 Nov 15;9:677. doi: 10.3389/fendo.2018.00677 (PMC6249314; doi:10.3389/fendo.2018.00677)
Supplement: Supplemental Table 12 — The top 20 genes from VEGAS2 gene-based analysis showing the strongest association with LDL-C level in typed GWAS data. [file Table_12.DOCX]

**Supplemental Table 12** The top 20 genes from VEGAS2 gene-based analysis showing the strongest association with LDL-C level in typed GWAS data

| Chr | Gene | Numbers of SNP | Start position | Stop position | Gene-based test statistic | Gene *P* value | Top-SNP | Top-SNP  *P* value |
| --- | --- | --- | --- | --- | --- | --- | --- | --- |
| 11 | *ANO1-AS2* | 4 | 69,918,539 | 69,921,454 | 45.16 | 1.26E-04 | rs4515996 | 2.15E-05 |
| 19 | *PVRL2* | 34 | 45,349,392 | 45,392,485 | 147.69 | 1.32E-04 | rs283810 | 2.33E-05 |
| 11 | *SRSF8* | 3 | 94,800,040 | 94,804,387 | 30.68 | 2.05E-04 | rs1056986 | 1.72E-04 |
| 6 | *TMEM14A* | 12 | 52,535,883 | 52,551,385 | 116.41 | 2.34E-04 | rs2670153 | 6.97E-05 |
| 1 | ***NTRK1*** | 38 | 156,785,541 | 156,851,642 | 160.52 | 4.49E-04 | rs1800879 | 1.12E-04 |
| 1 | ***FAF1*** | 78 | 50,906,934 | 51,425,936 | 523.51 | 5.25E-04 | rs3789587 | 1.51E-04 |
| 8 | *TRMT12* | 3 | 125,463,047 | 125,465,266 | 18.09 | 5.71E-04 | rs3812475 | 2.61E-03 |
| 1 | *ADAMTSL4* | 7 | 150,521,844 | 150,533,412 | 34.59 | 5.72E-04 | rs76075180 | 9.04E-04 |
| 12 | *KRT86* | 7 | 52,695,648 | 52,702,947 | 56.39 | 5.76E-04 | rs12832364 | 9.40E-04 |
| 23 | *SPANXA2-OT1* | 10 | 140,590,842 | 140,738,069 | 85.79 | 5.77E-04 | rs5954382 | 2.94E-04 |
| 16 | ***SNTB2*** | 22 | 69,221,049 | 69,342,955 | 111.36 | 6.34E-04 | rs34096150 | 5.80E-04 |
| 23 | *GPR112* | 12 | 135,383,121 | 135,499,047 | 87.69 | 6.67E-04 | rs4829829 | 8.53E-05 |
| 2 | *REG1B* | 3 | 79,312,148 | 79,315,150 | 25.39 | 6.71E-04 | rs3739144 | 1.39E-03 |
| 2 | *LOC643387* | 3 | 239,140,326 | 239,142,985 | 34.13 | 7.61E-04 | rs111384196 | 7.10E-04 |
| 11 | *PRSS23* | 5 | 86,511,490 | 86,522,273 | 39.27 | 7.70E-04 | rs2155080 | 3.24E-04 |
| 19 | *ZNF676* | 5 | 22,361,902 | 22,379,753 | 40.21 | 7.71E-04 | rs11666326 | 1.13E-03 |
| 7 | *WBSCR27* | 5 | 73,248,920 | 73,256,855 | 35.71 | 7.77E-04 | rs13246460 | 1.18E-03 |
| 1 | *APH1A* | 3 | 150,237,798 | 150,241,609 | 17.52 | 8.87E-04 | rs2275778 | 1.01E-04 |
| 18 | *ADNP2* | 14 | 77,866,914 | 77,898,228 | 70.40 | 9.00E-04 | rs11661201 | 4.98E-04 |
| 3 | *TMPRSS7* | 28 | 111,758,464 | 111,800,116 | 139.61 | 9.07E-04 | rs9288935 | 4.50E-04 |

**Note**: Chr, chromosome; The content discussed in detail were in bold.
