# Supplementary material for: Heritability and Genome-Wide Association Study of Plasma Cholesterol in Chinese Adult Twins
Source: Front Endocrinol (Lausanne). 2018 Nov 15;9:677. doi: 10.3389/fendo.2018.00677 (PMC6249314; doi:10.3389/fendo.2018.00677)
Supplement: Supplemental Table 13 — Common genes among TC, HDL-C and LDL-C level (P < 0.05). [file Table_13.DOCX]

**Supplemental Table 13** Common genes among TC, HDL-C and LDL-C level (*P* < 0.05)

| Gene symbol | *P* for TC level | *P* for HDL-C level | *P* for LDL-C level |
| --- | --- | --- | --- |
| ***FAF1*** | 3.26E-04 | 3.78E-03 | 5.25E-04 |
| *PRSS23* | 4.13E-04 | 2.53E-02 | 7.70E-04 |
| *TMPRSS7* | 8.37E-04 | 3.23E-02 | 9.07E-04 |
| *TRIM33* | 9.30E-04 | 9.40E-03 | 1.58E-03 |
| *KCNV2* | 2.05E-04 | 1.80E-02 | 1.76E-03 |
| *RFPL3S* | 4.98E-03 | 4.95E-02 | 3.15E-03 |
| *FXN* | 4.08E-04 | 3.40E-02 | 3.74E-03 |
| *LOC148709* | 1.56E-03 | 4.65E-02 | 3.80E-03 |
| *CDKN2C* | 4.60E-03 | 3.18E-02 | 4.13E-03 |
| *DDX31* | 8.95E-03 | 9.30E-03 | 6.05E-03 |
| *ZNF275* | 2.63E-02 | 7.24E-03 | 7.21E-03 |
| *LOC101927583* | 2.32E-04 | 6.37E-04 | 8.64E-03 |
| *SERPINA3* | 2.50E-02 | 3.95E-02 | 9.18E-03 |
| *PTPLA* | 6.05E-03 | 1.56E-02 | 9.19E-03 |
| *DOLPP1* | 3.60E-03 | 1.90E-02 | 9.50E-03 |
| *MYBBP1A* | 2.82E-03 | 1.04E-02 | 1.05E-02 |
| *ZNF536* | 6.08E-03 | 9.14E-03 | 1.07E-02 |
| *C7orf49* | 2.67E-03 | 2.00E-02 | 1.22E-02 |
| *ZNF586* | 4.28E-03 | 1.08E-02 | 1.22E-02 |
| *C16orf71* | 1.25E-02 | 4.12E-02 | 1.34E-02 |
| *MOAP1* | 6.65E-03 | 1.98E-02 | 1.40E-02 |
| *KCNIP3* | 7.61E-03 | 4.07E-02 | 1.41E-02 |
| *PODXL2* | 1.23E-02 | 2.10E-02 | 1.45E-02 |
| *NR5A1* | 1.90E-02 | 2.23E-02 | 1.46E-02 |
| *CCL22* | 1.73E-03 | 2.43E-02 | 1.57E-02 |
| *HECTD3* | 2.49E-04 | 4.19E-04 | 1.60E-02 |
| *YY1* | 2.91E-02 | 1.91E-02 | 1.64E-02 |
| *NR6A1* | 4.26E-02 | 2.76E-02 | 1.68E-02 |
| *CD1E* | 3.11E-04 | 1.69E-02 | 1.70E-02 |
| *LOC442028* | 3.63E-03 | 3.01E-02 | 1.70E-02 |
| ***KLKB1*** | 5.26E-04 | 7.10E-05 | 1.70E-02 |
| *IFIT5* | 1.35E-02 | 1.93E-02 | 1.73E-02 |
| *TYW5* | 3.22E-03 | 8.64E-03 | 1.87E-02 |
| *IL1RAPL2* | 8.23E-03 | 4.42E-02 | 1.90E-02 |
| *CRYBB1* | 2.58E-02 | 8.32E-03 | 1.92E-02 |
| *PLCZ1* | 4.53E-02 | 2.59E-02 | 1.99E-02 |
| *LOC101928417* | 1.75E-02 | 1.74E-02 | 2.02E-02 |
| *SLC26A10* | 2.59E-04 | 2.90E-05 | 2.13E-02 |
| *LOC101927666* | 2.90E-03 | 3.31E-02 | 2.20E-02 |
| *NCK2* | 1.91E-02 | 1.34E-02 | 2.40E-02 |
| *PROM2* | 3.57E-02 | 4.53E-02 | 2.51E-02 |
| *UROD* | 4.84E-04 | 7.57E-04 | 2.54E-02 |
| *CDK2* | 5.77E-03 | 1.02E-02 | 2.56E-02 |
| *CDV3* | 1.67E-02 | 6.03E-03 | 2.67E-02 |
| *MEX3B* | 9.80E-04 | 9.40E-03 | 2.93E-02 |
| *LRRTM1* | 1.76E-03 | 8.02E-03 | 3.03E-02 |
| *TSKU* | 2.81E-02 | 2.11E-02 | 3.09E-02 |
| *WDR41* | 1.40E-03 | 3.81E-03 | 3.35E-02 |
| *CTD-2151A2.1* | 1.88E-02 | 3.72E-02 | 3.39E-02 |
| *SPTBN2* | 5.62E-03 | 2.98E-02 | 3.40E-02 |
| *PPP2R3A* | 1.31E-03 | 4.03E-02 | 3.51E-02 |
| *OS9* | 3.97E-03 | 1.09E-03 | 3.51E-02 |
| *RNF214* | 4.80E-03 | 3.21E-02 | 3.52E-02 |
| *CABP1* | 3.87E-03 | 2.13E-02 | 3.60E-02 |
| *SNAPC2* | 1.78E-02 | 4.37E-02 | 3.69E-02 |
| *TEAD4* | 1.17E-03 | 9.00E-03 | 3.73E-02 |
| *CHST13* | 1.82E-02 | 4.15E-02 | 3.93E-02 |
| *FAM206A* | 4.59E-02 | 3.33E-02 | 4.06E-02 |
| *CAPN9* | 4.49E-02 | 3.32E-02 | 4.11E-02 |
| *ZNF556* | 4.51E-02 | 3.72E-03 | 4.21E-02 |
| *TIAL1* | 3.29E-02 | 4.23E-02 | 4.30E-02 |
| *RFT1* | 3.24E-02 | 9.90E-03 | 4.33E-02 |
| *PTTG1IP* | 1.25E-02 | 2.84E-02 | 4.37E-02 |
| *ZNF250* | 4.70E-02 | 4.46E-02 | 4.47E-02 |
| *TTC32* | 3.71E-02 | 2.96E-02 | 4.58E-02 |
| *B3GNT7* | 1.73E-02 | 4.88E-02 | 4.60E-02 |
| *LPGAT1* | 1.42E-03 | 3.95E-02 | 4.72E-02 |
| *PPL* | 1.20E-02 | 1.37E-02 | 4.75E-02 |
| *JPH3* | 2.81E-03 | 2.60E-02 | 4.76E-02 |
| *ZNF154* | 2.72E-02 | 1.34E-02 | 4.85E-02 |
| *GAS6-AS2* | 1.11E-02 | 2.53E-03 | 4.87E-02 |

The content discussed in detail were in bold.
