# Supplementary material for: Heritability and Genome-Wide Association Study of Plasma Cholesterol in Chinese Adult Twins
Source: Front Endocrinol (Lausanne). 2018 Nov 15;9:677. doi: 10.3389/fendo.2018.00677 (PMC6249314; doi:10.3389/fendo.2018.00677)
Supplement: Supplemental Table 14 — The top 20 pathway results-KEGG, Reactome, and Biocarta (emp-P < 0.05) using PASCAL program for TC level in typed GWAS data. [file Table_14.DOCX]

**Supplemental Table14** The top 20 pathway results-KEGG, Reactome, and Biocarta (emp-*P* value < 0.05) using PASCAL program for TC level in typed GWAS data

| Pathway | chisq-*P* | emp-*P* | -log(chisq*P*) | -log(emp*P*) |
| --- | --- | --- | --- | --- |
| REACTOME_INTRINSIC_PATHWAY | 4.17E-04 | 3.49E-04 | 3.38001 | 3.45717 |
| REACTOME_FORMATION_OF_FIBRIN_CLOT_CLOTTING_CASCADE | 4.17E-04 | 3.51E-04 | 3.38001 | 3.45469 |
| REACTOME_MEIOSIS | 3.60E-03 | 5.60E-04 | 2.44311 | 3.25181 |
| REACTOME_NEPHRIN_INTERACTIONS | 1.86E-03 | 5.80E-04 | 2.73125 | 3.23657 |
| REACTOME_E2F_MEDIATED_REGULATION_OF_DNA_REPLICATION | 2.02E-03 | 8.40E-04 | 2.69522 | 3.07572 |
| REACTOME_**ZINC_TRANSPORTERS** | 1.31E-03 | 1.19E-03 | 2.88309 | 2.92445 |
| REACTOME_**METAL_ION_SLC_TRANSPORTERS** | 1.31E-03 | 1.19E-03 | 2.88309 | 2.92445 |
| REACTOME_INHIBITION_OF_REPLICATION_INITIATION_OF_DAMAGED  _DNA_BY_RB1_E2F1 | 1.26E-03 | 1.21E-03 | 2.89982 | 2.91721 |
| REACTOME_PLATELET_SENSITIZATION_BY_LDL | 1.26E-03 | 1.23E-03 | 2.89982 | 2.91009 |
| REACTOME_CELL_CELL_COMMUNICATION | 1.71E-03 | 1.33E-03 | 2.76636 | 2.87615 |
| REACTOME_**ERKS_ARE_INACTIVATED** | 1.26E-03 | 1.38E-03 | 2.89982 | 2.86012 |
| REACTOME_CTNNB1_PHOSPHORYLATION_CASCADE | 1.26E-03 | 1.40E-03 | 2.89982 | 2.85387 |
| REACTOME_**MAPK_TARGETS_NUCLEAR_EVENTS_MEDIATED**  **_BY_MAP_KINASES** | 1.26E-03 | 1.44E-03 | 2.89982 | 2.84164 |
| REACTOME_NUCLEAR_EVENTS_KINASE_AND_TRANSCRIPTION  _FACTOR_ACTIVATION | 1.26E-03 | 1.48E-03 | 2.89982 | 2.82974 |
| REACTOME_**ERK_MAPK_TARGETS** | 1.26E-03 | 1.50E-03 | 2.89982 | 2.82391 |
| REACTOME_**TRANSPORT_OF_GLUCOSE_AND_OTHER_SUGARS_BILE_SALTS**  **_AND_ORGANIC_ACIDS_METAL_IONS_AND_AMINE_COMPOUNDS** | 1.31E-03 | 1.51E-03 | 2.88309 | 2.82102 |
| REACTOME_CELL_JUNCTION_ORGANIZATION | 1.91E-03 | 1.62E-03 | 2.71879 | 2.79048 |
| BIOCARTA_AHSP_PATHWAY | 1.76E-03 | 1.90E-03 | 2.75549 | 2.72125 |
| REACTOME_APOPTOTIC_CLEAVAGE_OF_CELL_ADHESION_PROTEINS | 2.20E-03 | 2.06E-03 | 2.65753 | 2.68613 |
| REACTOME_**AMINO_ACID_TRANSPORT_ACROSS_THE_PLASMA_MEMBRANE** | 4.18E-03 | 2.17E-03 | 2.37915 | 2.66354 |

**Note**: chisq-*P*, Chi-square *p* value. Chi-squared method (gene-score *p*-value were ranked and transformed to a uniform distribution, these values were then transformed by a chi-square quantile function, and summed).

emp-*P*, empirical *p* value. Empirical sampling method (gene-scores are transformed with chi-square quantile function and summed, then Monte Carlo estimate of the *p* values were obtained by sampling random sets of the same size).

The content discussed in detail were in bold.
