# Supplementary material for: Heritability and Genome-Wide Association Study of Plasma Cholesterol in Chinese Adult Twins
Source: Front Endocrinol (Lausanne). 2018 Nov 15;9:677. doi: 10.3389/fendo.2018.00677 (PMC6249314; doi:10.3389/fendo.2018.00677)
Supplement: Supplemental Table 15 — The top 20 pathway results-KEGG, Reactome, and Biocarta (emp-P < 0.05) using PASCAL program for LDL-C level in typed GWAS data. [file Table_15.DOCX]

**Supplemental table 15** The top 20 pathway results-KEGG, Reactome, and Biocarta (emp-*P* value < 0.05) using PASCAL program for LDL-C level in typed GWAS data

| Pathway | chisq-*P* | emp-*P* | -log(chisq*P*) | -log(emp*P*) |
| --- | --- | --- | --- | --- |
| KEGG_**FC_EPSILON_RI_SIGNALING_PATHWAY** | 7.53E-04 | 1.41E-04 | 3.12301 | 3.85078 |
| KEGG_COLORECTAL_CANCER | 1.02E-03 | 2.06E-04 | 2.99332 | 3.68613 |
| REACTOME_MEIOSIS | 1.18E-03 | 2.17E-04 | 2.92838 | 3.66354 |
| BIOCARTA_CCR5_PATHWAY | 1.38E-03 | 3.17E-04 | 2.85881 | 3.49894 |
| BIOCARTA_TCR_PATHWAY | 1.74E-03 | 4.13E-04 | 2.75985 | 3.38405 |
| BIOCARTA_FCER1_PATHWAY | 1.74E-03 | 4.50E-04 | 2.75985 | 3.34679 |
| BIOCARTA_**NFAT_PATHWAY** | 1.74E-03 | 5.60E-04 | 2.75985 | 3.25181 |
| BIOCARTA_AGR_PATHWAY | 2.12E-03 | 7.10E-04 | 2.67336 | 3.14874 |
| REACTOME_**METABOLISM_OF_AMINO_ACIDS_AND_DERIVATIVES** | 1.32E-03 | 7.60E-04 | 2.87795 | 3.11919 |
| KEGG_**ERBB_SIGNALING_PATHWAY** | 2.82E-03 | 7.70E-04 | 2.54923 | 3.11351 |
| BIOCARTA_ATM_PATHWAY | 1.13E-03 | 8.10E-04 | 2.94743 | 3.09151 |
| BIOCARTA_BCR_PATHWAY | 2.66E-03 | 8.30E-04 | 2.57581 | 3.08092 |
| REACTOME_MEIOTIC_SYNAPSIS | 3.05E-03 | 8.70E-04 | 2.51638 | 3.06048 |
| REACTOME_CELL_JUNCTION_ORGANIZATION | 1.65E-03 | 9.00E-04 | 2.78181 | 3.04576 |
| BIOCARTA_**IL1R_PATHWAY** | 3.16E-03 | 9.30E-04 | 2.50047 | 3.03152 |
| BIOCARTA_CERAMIDE_PATHWAY | 1.13E-03 | 9.40E-04 | 2.94743 | 3.02687 |
| BIOCARTA_STRESS_PATHWAY | 3.16E-03 | 9.50E-04 | 2.50047 | 3.02228 |
| KEGG_EPITHELIAL_CELL_SIGNALING_IN_HELICOBACTER  _PYLORI_INFECTION | 3.16E-03 | 9.60E-04 | 2.50047 | 3.01773 |
| KEGG_PANCREATIC_CANCER | 3.16E-03 | 9.70E-04 | 2.50047 | 3.01323 |
| REACTOME_**HIV_INFECTION** | 1.35E-03 | 9.80E-04 | 2.86868 | 3.00877 |

**Note**: chisq-*P*, Chi-square *p* value. Chi-squared method (gene-score *p*-value were ranked and transformed to a uniform distribution, these values were then transformed by a chi-square quantile function, and summed).

emp-*P*, empirical *p* value. Empirical sampling method (gene-scores are transformed with chi-square quantile function and summed, then Monte Carlo estimate of the *p* values were obtained by sampling random sets of the same size).

The content discussed in detail were in bold.
